# Atomistic insight into salinity dependent preferential binding of polar aromatics to calcite/brine interface: implications to low salinity waterflooding

**DOI:** 10.1038/s41598-021-91402-0

**Published:** 2021-06-07

**Authors:** Mohammad Mehdi Koleini, Mohammad Hasan Badizad, Hassan Mahani, Ali Mirzaalian Dastjerdi, Shahab Ayatollahi, Mohammad Hossein Ghazanfari

**Affiliations:** Department of Chemical and Petroleum Engineering, Sharif University of Technology, Tehran, Iran

**Keywords:** Solid Earth sciences, Chemistry, Energy science and technology, Engineering

## Abstract

This paper resolve the salinity-dependent interactions of polar components of crude oil at calcite-brine interface in atomic resolution. Molecular dynamics simulations carried out on the present study showed that ordered water monolayers develop immediate to a calcite substrate in contact with a saline solution. Carboxylic compounds, herein represented by benzoic acid (BA), penetrate into those hydration layers and directly linking to the calcite surface. Through a mechanism termed screening effect, development of hydrogen bonding between –COOH functional groups of BA and carbonate groups is inhibited by formation of a positively-charged Na^+^ layer over CaCO_3_ surface. Contrary to the common perception, a sodium-depleted solution potentially intensifies surface adsorption of polar hydrocarbons onto carbonate substrates; thus, shifting wetting characteristic to hydrophobic condition. In the context of enhanced oil recovery, an ion-engineered waterflooding would be more effective than injecting a solely diluted saltwater.

## Introduction

The nanoscale interactions at the contact of solid/fluid interfaces critically determine characteristics at the surface of different minerals^1–5^. In this play, Ions take the key role, dominantly contributing to the interplay of water-calcite-hydrocarbon^6–8^. Interestingly, wetting state of naturally occurring minerals, termed as wettability, is an ion-specific property, which could be effectively modified via manipulating ionic content of surrounding brine solutions^9–11^. This virtue is central to an operation in the oil industry for enhanced oil recovery (EOR), mostly using water with different salinities.

Before oil migration into the pores of rocks, the surface of reservoirs has been wetted by high salinity brine, termed as formation water^12,13^. Since the last two decades, laboratory and field experiences have proven the effectiveness of applying low salinity waterflooding (LSW), controlled/engineered salinity waterflooding and smart water techniques in order to improve oil recovery from carbonate rocks^14–19^. LSW is an enhanced oil recovery (EOR) technique which involves injecting diluted or ionically modified brine into carbonate oil reservoirs. The injection of low salinity brine into the reservoirs disturbs the long standing thermodynamic equilibrium at mineral/formation water interface^20^. Such disturbances complicate the governing mechanisms of oil/brine/rock interactions and is the reason behind the uncertainties dealing with LSW^14,17,21^. Due to this complexity, it is believed that recognition of a combination of mechanisms rather than a single one is more logical^17,21–28^.

It should be emphasized that calcite (CaCO_3_) is one of the main constituting minerals of subsurface petroleum resources^29–31^. Despite vast progress in the empirical lab-scale research, the atomic-level interactions behind the salinity-response of calcite minerals is still not well understood. Koleini et al^32^ recently showed that salinity-dependence of calcite wettability is essentially driven by adsorption/desorption process. They observed stronger surface attachment of polar hydrocarbons, represented there by benzoate, upon increasing salinities. However, polar compounds in crude oil do not entirely ionize in the brine solution, while bearing in mind that subsurface reservoirs are typically at neutral pH condition, 6.5–7.5 in carbonates^33–35^. It is natural to expect different salinity response for the interaction of carboxylic acids with calcite compared to ionized molecules. It seems that the complexity of crude oil is partly the origin of inconsistent observations obtained in experiments concerning brine injection^36^. Moreover, the presence of ions in brine is also a matter of interest which intensifies the complexity of calcite/oil/brine system^37^. The occupation of available sites at calcite/brine interface by electrolyte ions can either drives the surface less or more water-wet depending on the ions^38,39^. Some of the impacts of ions include ion-exchange^37,40^, pinning point^41^ and screening effect^42^ which diversely alter rock wettability.

Despite numerous empirical and theoretical studies on LSW in the last two decades, the driving mechanism(s) of that process is not well understood^12^. Most suggested mechanisms are somehow connected with solid–liquid interface. Recent publications have placed emphasis on the necessity of hierarchical multi-scale inspections, from atomic to field scale, for characterizing mineral/oil/water interfaces^19,43^. Within that spectrum of research, molecular-level study lays the foundation for ascertaining behavior of ions and their interplay with organic compounds in the brine films covering pore wall of oil formations^12^. Hence, a molecular-level understanding is expected to deepen our knowledge of different complex nanoscale interactions between calcite and oil components upon brine injection. Atomistic simulation is a robust technique for examining contribution and interplay of compounds at interface media. This modelling approach has recently been attracted wide interest both in science and technology. For instance, in a recent paper by Wang et al., the microscopic origin of polymer matrix reinforcement by nanodiamonds was investigated by applying classical molecular dynamics (MD) simulations^44^. There has been an increasing growth in the molecular-level investigations concerning enhanced oil recovery development. For example, in a recent paper by Fang et al., the oil swelling and detachment mechanisms through gas injection to deep reservoirs was explored using MD simulations^45^.

To remove such uncertainties, atomistic simulation affords strong capability for examining the contribution of ions to the interfacial characteristics of minerals with sub-nanoscale resolution. Simulations have revealed that ions can either act as mediating agents to develop hydrocarbon-calcite interactions^41,46–48^ or efficiently screen the attachment of hydrocarbons onto the surface^42,49^. While calcite turns into weakly water-wet state in the former condition, water-favoring character of the surface is advanced upon the screening by ions. Hence, the net result of these wettability alterations depends on the composition of the brine and oil components. Molecular simulation affords unique opportunity towards a systematic examination of individual function of leading agents of oil/brine/rock system governing surface wettability.

In this study, we performed MD simulation to disclose the affinity of a model oil adsorption (mimicked by benzoic acid, BA, as a generic neutral aromatic carboxylic acid) onto calcite at different brine concentrations. The focus of the work is on the influence of ionic content and salinity level of the NaCl brine on surface wettability. The distribution of ions at solid/liquid interface would provide an insight into the likely dominant mechanisms governing the interactions of calcite/oil/brine system also verified by quantum calculations.

The paper has been laid out as follows: first, we describe the simulation methodology with a concise explanation concerning the necessity of individual constituents of the simulation system. Then, we present the results of our MD simulations which include density profiles, distribution functions, vertical/planar trajectory patterns, displacement and diffusivity behavior of species and adsorption energetic terms of components. Finally, the results and discussions are summarized in the conclusion section. We believe that fundamental studies like this can provide insights into underlying mechanisms concerning the role of ions at calcite/brine interface, their arrangement and how this is altered by lowering brine salinity.

## Methods

### Molecular dynamics simulation

We performed MD simulations to find the affinity of polar hydrocarbons to adsorb at calcite/water interface in NaCl brines of different salinities. The calcite slab is terminated by [1014] plane because this low index surface has been proved to be the most stable cleavage plane of the mineral^50–52^. We ignored mineral dissolution in our simulations based on the experimental findings of Romanuka et al^53^, Mahani et al^16^ and Nasralla et al^54,55^ where rock dissolution is believed not to be the primary mechanism in calcite wettability alteration. In our simulations, we focus on the impact of Na^+^ and Cl^−^ ions which are expected to display the dominant role in preferred wettability of calcite since these are the main ionic constituents of sea and connate water^56–58^. We believe that the role of NaCl in the experimental studies has been underestimated.

The facile accessibility of seawater (with a commonly observed salinity in the range of 30,000–60,000 ppm^17,36,59^) makes it an alternative to formation water (which can be as high as 200,000 ppm^36,60^) for water injection techniques utilized in oil recovery process. In this regard, four aqueous solution containing Na^+^ and Cl^−^ ions were considered in the present study: deionized water (DW), dilute seawater (dSW; 30,000 ppm), seawater (SW; 60,000 ppm) and high salinity brine (HS; 210,000 ppm). Benzoic acid (BA), as a simple aromatic polar hydrocarbon with enough solubility in water, is selected as a proper candidate to simultaneously mimic both polar and aromatic components of crude oil which mostly include carboxylic acids, asphaltenes and resins^61^. In this manner, the main focus in this study is to specifically explore the impact of salinity on the adsorption of neutral polar compounds, as a constituent in brine, onto calcite surface.

The calcite slab dimensions are 40.49 Å × 39.93 Å × 16.94 Å, while the surface normal is in the z-direction. Our preliminary evaluation verified the system is large enough to preclude any potential artifact due to finite-size effect. The benchmarking process is detailed in section S1 the Supplementary Information.

In each simulation, 15 BA molecules were randomly placed in the middle of the simulation box at least 10 Å above the mineral with adequate number of ions to achieve desired salinity. Then, the empty space of the simulation box was filled in by 1350 water molecules which nearly satisfied the normal density of water, i.e. 1 gr cm^−3^. The initial configurations were constructed by means of the graphical user interface (GUI) implemented as a tool in Dl_poly package. A typical initial configuration of the ensemble is shown in Figure S1 of Supplementary Information with further details. Throughout, the calcium and carbon atoms of the calcite slab were constrained to their initial positions in order to avoid possible distortion or non-uniformity of the calcite/water interface during the simulation. By doing so, the calcite slab is considered insoluble which is in accord with thermodynamic and kinetic characteristics of CaCO_3_, which barely and slowly dissolves in water^62–64^. Moreover, atomic force microscopy performed by Ricci et al^65^ revealed the smooth nano-surface of CaCO_3_ contacting aqueous solutions.

The periodic boundary conditions were applied in all directions and meanwhile a 5 nm-length vacuum space was placed above the fluid phase to avoid any unwanted interaction of period images along the z-direction. The van der Waals and electronic contributions are respectively described by Lennard-Jones (LJ) 12-6 and Coulomb potentials. The forcefield parameterization from Shen et al^66^ was applied for calcite which was found to be compatible with SPC/E^67^ model for water and Smith and Dang^68^ parameters for ions used in our study. We used the OPLS-AA^69^ forcefield to describe atomwise parameters for BA. The cross nonbonded interactions were determined by applying Lorentz–Berthelot mixing rule from atomwise LJ parameters. All forcefield parameters taken in this study are reported in Table S1 of SI. The simulations were performed with the NVT ensemble with a time step of 1 fs for a total simulation time of 20 ns after 2 ns of equilibration at the beginning of the simulation. The data from the last 10 ns of the simulation were gathered for statistical analysis at every 2 fs interval. Adequacy of the simulation timespan was evaluated on the basis of the time-evolution of MSD diagram (discussed later) and total energy of the system (Figure S5, Supplementary Information). It is evident that energy of all calcite/brine systems steadily transits to a constant minimum value within the initial 2 ns.

The temperature was set to 300 K by the Nosé–Hoover thermostat^70,71^ and long-range electrostatic interactions were computed by means of Ewald summation method with a precision of 10^−6^. A cut-off distance of 15 Å was assigned for LJ interactions. The simulations in this study were all performed with Dl_poly package^72^.

### Density functional theory

We also performed concise density functional theory (DFT) calculations as implemented in the Quantum Espresso software package by means of a Plane-Wave Self-Consistent Field (PWSCF) method to verify charge distribution over calcite surface in the presence of brine and BA. We used a (2 × 2) surface unit cell of [1014] calcite cleavage plane with two layers to study the adsorption of water, ions and BA. Twelve water molecules and 2 ion pairs of Na^+^/Cl^−^ were placed on a calcite supercell with dimensions of 8.095 × 9.98 × 18.42 Å^3^. The simulations were performed by adopting periodic boundary conditions as if they occur in an infinite space. More details are presented in Supplementary Information.

## Results and discussion

In this section, the micro-properties of the calcite/brine interface have been scrutinized and compared at varying salinity levels in terms of various analyses: density distribution diagrams, charge density profiles, angle distribution map, mean-square displacement (MSD), self-diffusivity coefficient, 2D (planar) density map, and vertical z-component trajectory of individual BA molecules. Furthermore, the adsorption isotherms of sodium and chloride ions have been provided to attain a quantitative insight into the impact of ions pairing (association) on extend of adsorption onto the CaCO_3_ surface.

### Density distribution diagram

**Figure 1:**
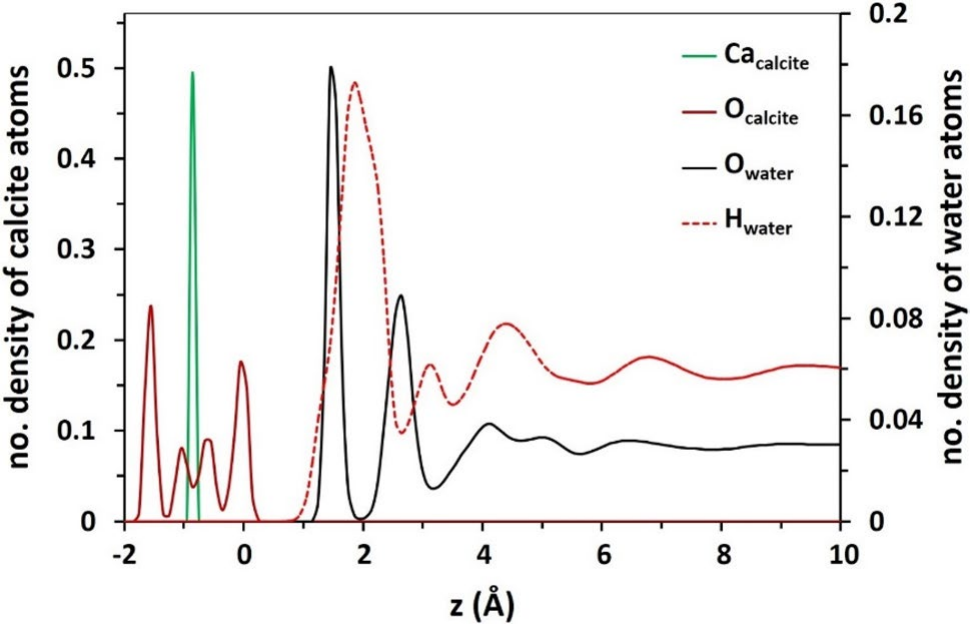
The density profile of water atoms with respect to Ca and O-atoms of the topmost calcite layer. These densities are almost identical in all salinities. The origin of the distance to surface measurements is based on the outermost O-atoms of calcite.

Density distribution plot is a useful analysis for assessing tendency of a certain compound (herein, water or ions) for accumulating near a solid/water interface compared to the bulk space. The density profiles of water atoms (O_w_ and H_w_) in Fig. 1 as a function of z, the axis normal to the surface, indicate that calcite surface is hydrated by a couple of highly-ordered water monolayers with maxima at 1.5 and 2.7 Å distance from the surface. These include a very compact solid-like monolayer of water directly covering the mineral followed by a lower density and slightly less ordered hydration layer in all salinities. The densities and thicknesses of these hydration layers are almost identical in all salinities. The nearly solid-like structure of the monolayer of water immediately over calcite validates the persisting wetness of the mineral^20^. Formation of two water mono-layers over a calcite surface are in agreement with X-ray reflectivity observations reported by Fenter et al^73^. Furthermore, it was recently shown that the formation of these well-structured hydration layers is triggered by the highly-ordered crystal structure of calcite^74^. The density profile of water over calcite is led by O_w_, which is in agreement with the observed direct binding of water via its oxygen atom to the outermost Ca atoms of calcite^75^.

While the distribution of water molecules at the beginning of simulation was random and statistically uniform within the simulation box, the strong hydrophilic nature of calcite surface perturbed the initial distribution of water molecules. Upon comparing density profiles of O_w_ and H_w_ obtained at successive timespans throughout the simulation, Figure S6, we notify the rapid development of wetting monolayers over the calcite substrate within the early 0.5 ns, revealing the prevailing interaction of water molecules with basal surface of the mineral substrate. Before the end of equilibration, density of water in proximity to surface reached a stable maximum which remained constant until the end of the simulation. The oscillations of water density beyond high density regions were also relieved before the end of equilibration and started to fluctuate around a constant value.

**Figure 2:**
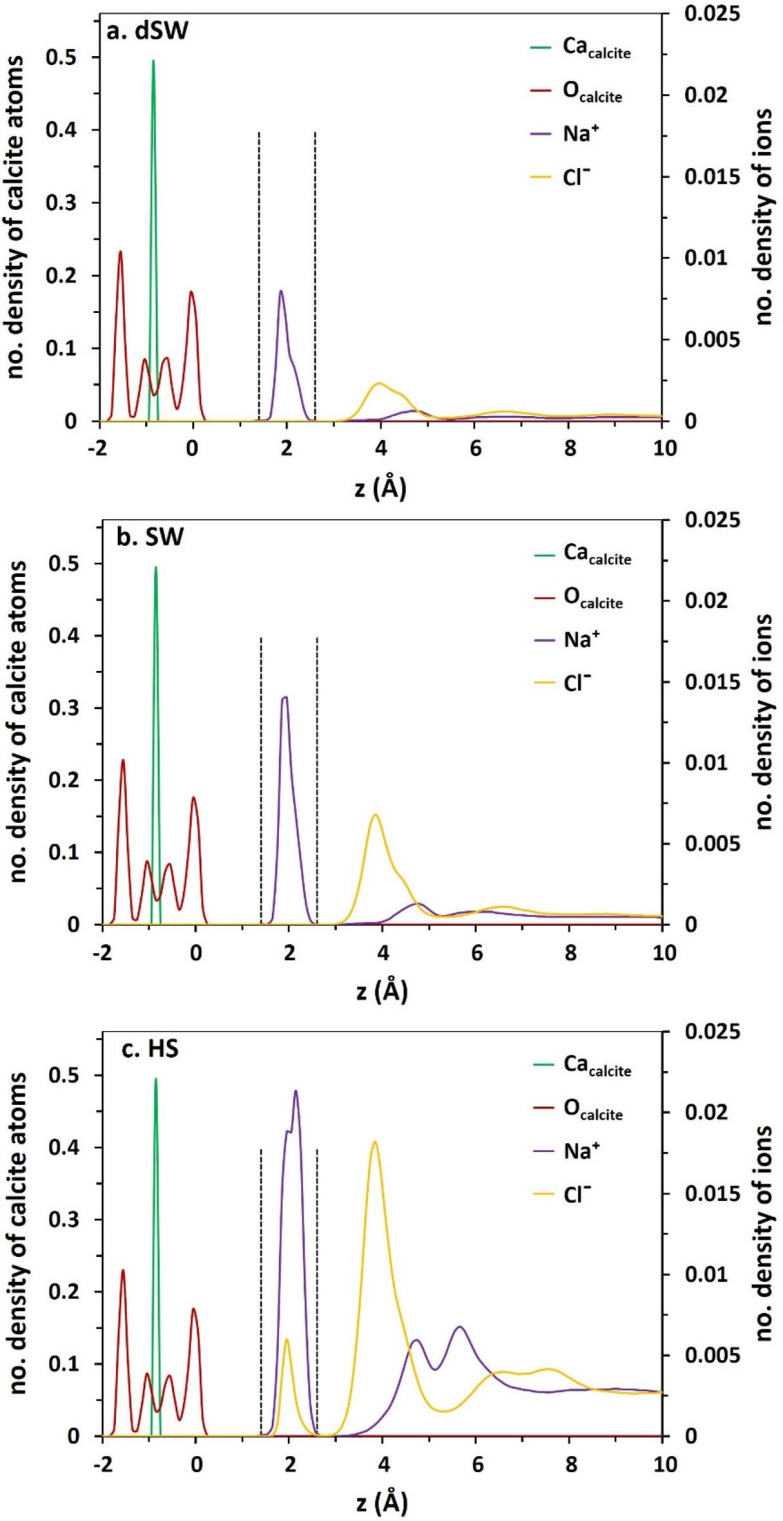
The density profile of ions in brines with respect to Ca and O-atoms of calcite topmost layer. The dashed lines highlight the position of water density maxima over surface.

**Figure 3:**
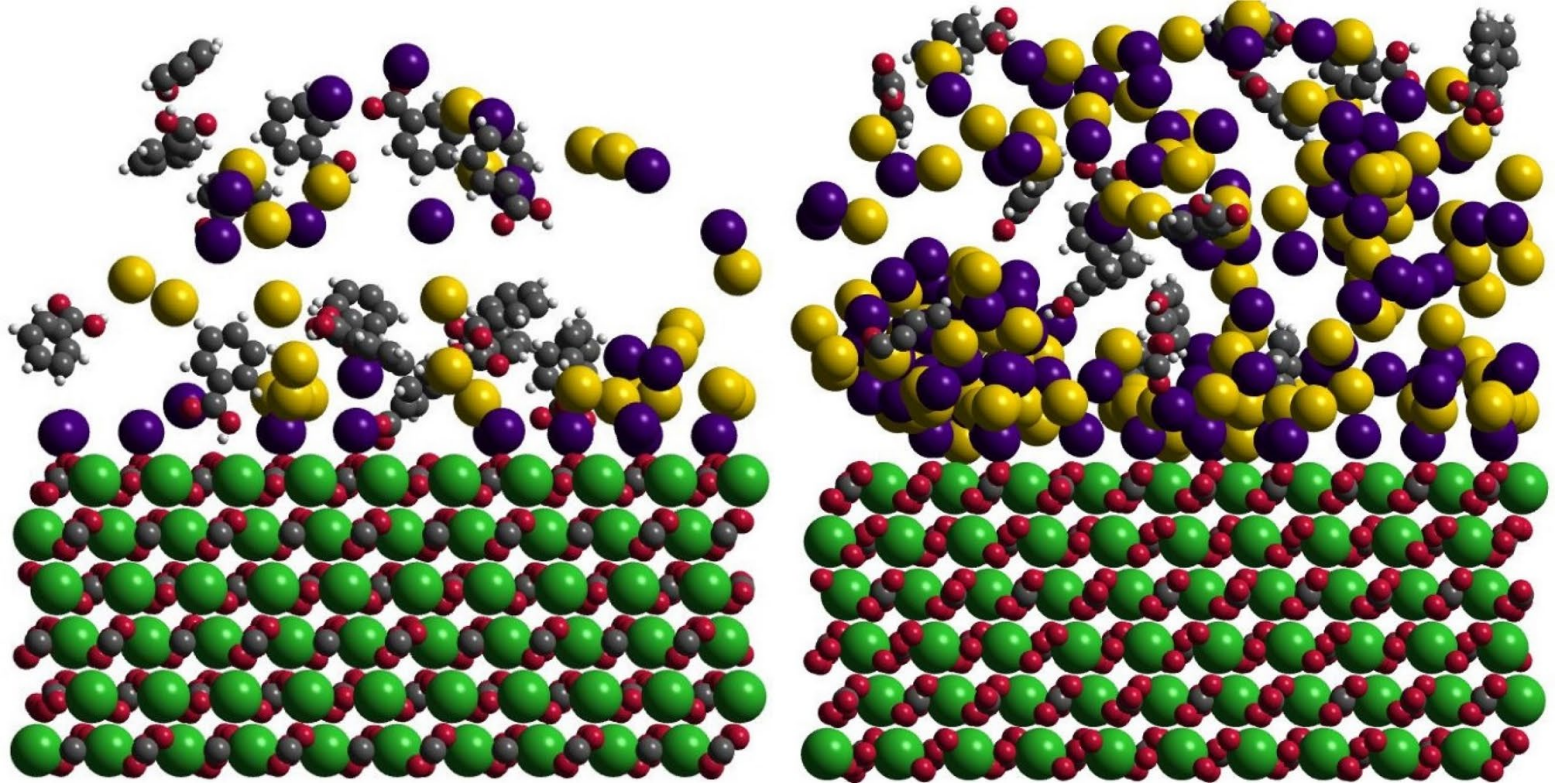
The final simulation snapshots of SW (left) and HS (right). The atoms/ions are drawn with the sizes of their radii. Water molecules are omitted for clarity. The same snapshots for DW and dSW are shown in Figure S3. The atom colors are: Ca; green, C; grey, O; red, H; white, Na; purple, Cl; yellow.

The interval between the position of the first peak of O_w_ density profile and the outermost Ca atoms of calcite, i.e. 2.3 Å observed in Fig. 1, is adequately reproduced by the position of the first shell of O_w_ around Ca atoms of the mineral calculated from radial distribution function (RDF) analysis (Figure S9, Supplementary Information). The slightly oscillating decreasing trend of water density beyond the two compact hydration layers reaches a smooth value approximately at 10 Å from the surface. This 1-nm length hydration layer above calcite is the basis of interface definition in our study. In brines, layered structure of Na^+^ and Cl^−^ is observed over calcite surface (Fig. 2a–c). The solid-like shape of Na^+^ density peak in adjacency to calcite, within the first compact hydration layer, suggests the strong direct binding of the cation to the substrate without any intervening water molecules. The appearance of a dense layer of Na^+^ ions between the peaks of first and second hydration layers might lead to a misinterpretation that the cation is not able to penetrate the first compact hydration layer. However, this fact must be considered that the preferential adsorption of Na^+^ onto calcite is over dangling O-atoms of the surface (the outermost O-atoms) while that of water is above Ca. This fact is supported by the AFM experiment performed by Ricci et al^65^, who directly visualized the preferential localization of Na^+^ cations on a calcite surface in contact with an electrolyte solution. The upper level of dangling oxygen compared to Ca in [1014] plane is the origin of the gap between the first maxima of water and Na^+^ peak next to the surface. Moreover, the compact shell of Na^+^ around O_calcite_ at 2.28 Å, verified by RDF calculations in Figure S10, closely conforms with the interval between the peaks of two atoms in density profiles (Fig. 2). This matching certifies the direct adsorption of sodium ions onto calcite via the basal (upper) O-atoms of the mineral. This is obviously seen from simulation snapshots in which the sodium ions are in fact in direct contact with dangling O-atoms of calcite (Fig. 3). On the other hand, Clˉ ions are localized above sodium ions beyond the hydration layers. Density profiles and simulation snapshots show that chlorides can only penetrate the compact monolayer of water in HS.

**Figure 4:**
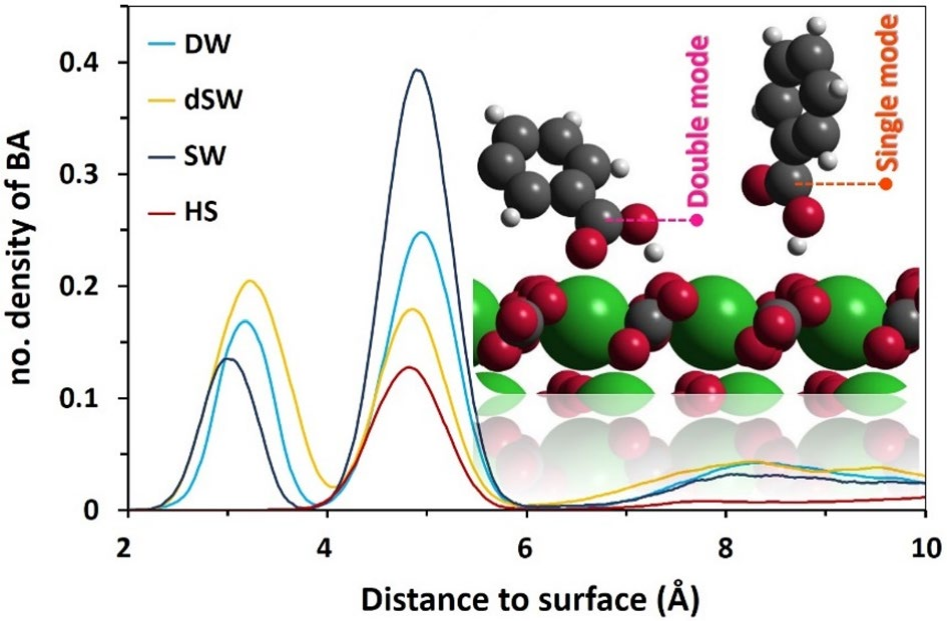
Density profile of BA over calcite surface. The peaks around 3 Å are the result of double mode interaction, while the ones nearby 5 Å are due to single mode. The inset is captured from simulation snapshots to represent two mode of interactions.

We tagged the C-atom of phenyl which links the ring to carboxylic functional group to represent the density profile of BA. The appearance of a couple of tight peaks of BA density in vicinity of the surface, observed within the compact hydration layers, is an evidence of two different modes of adsorption of the acid molecules at calcite/water interface (Fig. 4). However, in HS brine, only one peak is observed which might be the result of loss of preferential adsorption sites for the acid. Moreover, the integral area of BA density profile is smaller in HS brine than other brines which is again due to the screening effect of ions that reduces BA adsorption onto calcite.

### Charge density profile

**Figure 5:**
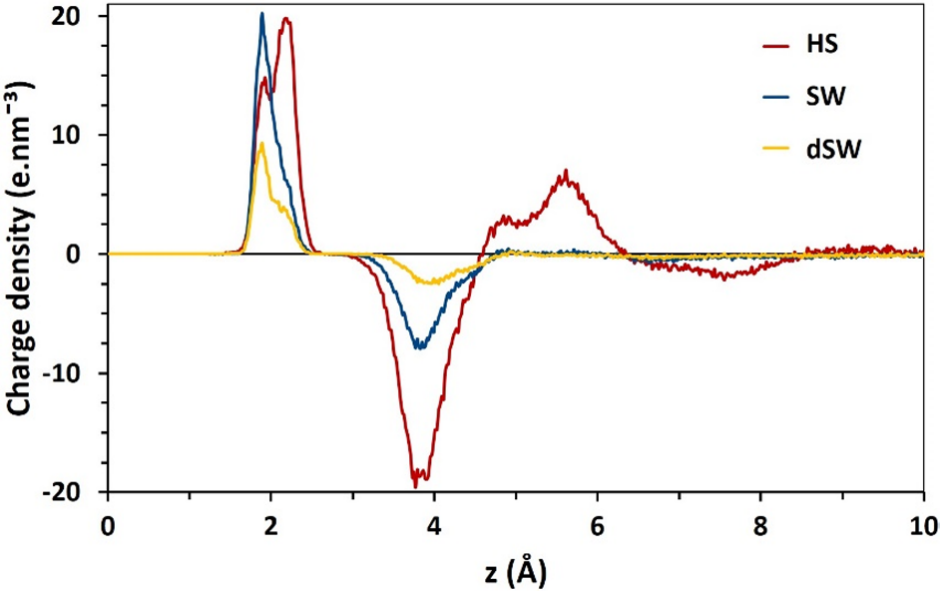
Charge density profile of ions over calcite surface at different brine salinities.

To deepen our knowledge on the ionic atmosphere in the vicinity of calcite surface, it is worth evaluating synchronous contribution of all ion types (Na^+^ and Cl^−^) on the basis of charge density profile. This way, we are able to ascertain existence and intensity of an electrical double layer (EDL) at mineral/brine interface. Charge density profiles in Fig. 5 reveal that ordered layering of ions over the surface leads to the formation of an EDL at calcite/brine interface. The protruding O-atoms of calcite, as the main reason of surface polarity, motivate the development of the EDL over the mineral by developing interaction with sodium ions. Successively, these layered sodium ions attract the negative chlorides toward the interface and thus the EDL is developed. Therefore, the intrinsic surface polarity causes the formation of the EDL in vicinity of a neutral surface which violates the prerequisite of the substrate to be charged for the development of EDL^47^. The positive layer of the EDL, composed of sodium ions, imitates a Stern layer while the negative one, made up of chlorides, is a diffuse-like layer^76,77^. Formation of salt layers over the mineral is expected to mediate BA-calcite interaction.

Unlike the common perception, there is no sign of double layer expansion in the present charge density profiles, shown in Fig. 5. This counter-intuitive observation arises by the particular charging mechanism of carbonates compared to metal oxide minerals, such as quartz (SiO_2_). In the latter, surface charge is developed via ionization of the basal silanol group (–SiOH), whereas the charging process in a CaCO_3_ substrate is induced by preferential adsorption of Na^+^ cations on the protruding oxygen atom of the basal carbonate groups. In this case, we deal with a Stern-like and diffuse-like charged mono-layers of sodium and chloride ions, respectively. This is clearly distinct from the conceptual Gouy–Chapman model where dilution renders larger thickness of the diffuse layer (known as Debye length)^78^.

Note that the effect of pH cannot be straightforwardly dealt with classical molecular simulation^79^. Furthermore, the commonly used force-fields developed for CaCO_3_ do not incorporate the impact of pH variation by assuming an intrinsically charge-neutralized mineral surface. This situation is indeed in accord with prevailing thermodynamic conditions of natural and lab-scale calcite-electrolyte systems, i.e., pH = 6–8^80^. The pH-dependent surface charge of carbonate minerals is yet controversial and its origin is not clearly understood^81^. At pH conditions beyond the iso-electric point, zeta potential measurements have pointed out the positively charged surface of carbonates^81^. Therefore, the accuracy and validity of MD results for EDL and associating discussion above is restricted to an iso-electric condition.

### Molecular orientation

**Figure 6:**
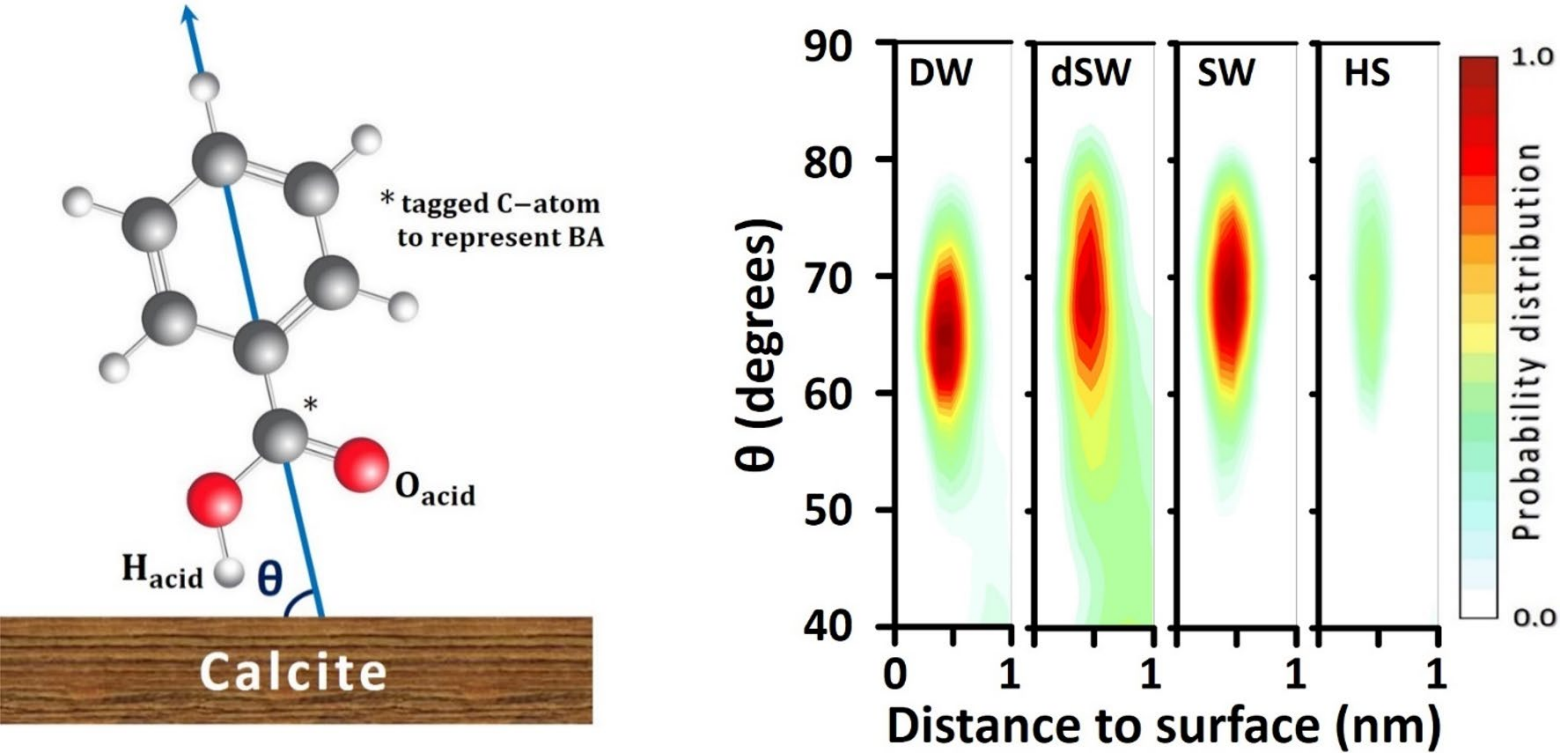
The schematic representation of inclination angle, θ, (left) and the distribution of θ over calcite surface (right).

The interplay between BA and calcite surface was further scrutinized by evaluating preferred orientation of those molecules at the mineral/brine interface. It helps us to figure out the most probable configuration whereby BA molecules interact with CaCO_3_ surface. For this purpose, Inclination angle, θ, was defined as the angle between calcite plane and the vector passing through phenyl ring and −COOH group. The probability distribution plot for inclination angle of BA molecules (Fig. 6) was obtained by tracking spatial position of each molecule (represented by z-component coordinate of the carbon atom in carboxylic group) within a set of conceptual bins parallel to the solid plane. The calculation process is explained in section S1 of Supplementary Information. In Fig. 6, θ is dominantly in the range of 60°–70°, verifying inclined orientation of BAs with the −COOH functional group toward calcite surface. Henceforth, the detailed analysis of the interaction between –COOH group and surface atoms would elaborately uncover the physical nature of BA adsorption onto calcite.

### Radial distribution function

**Figure 7:**
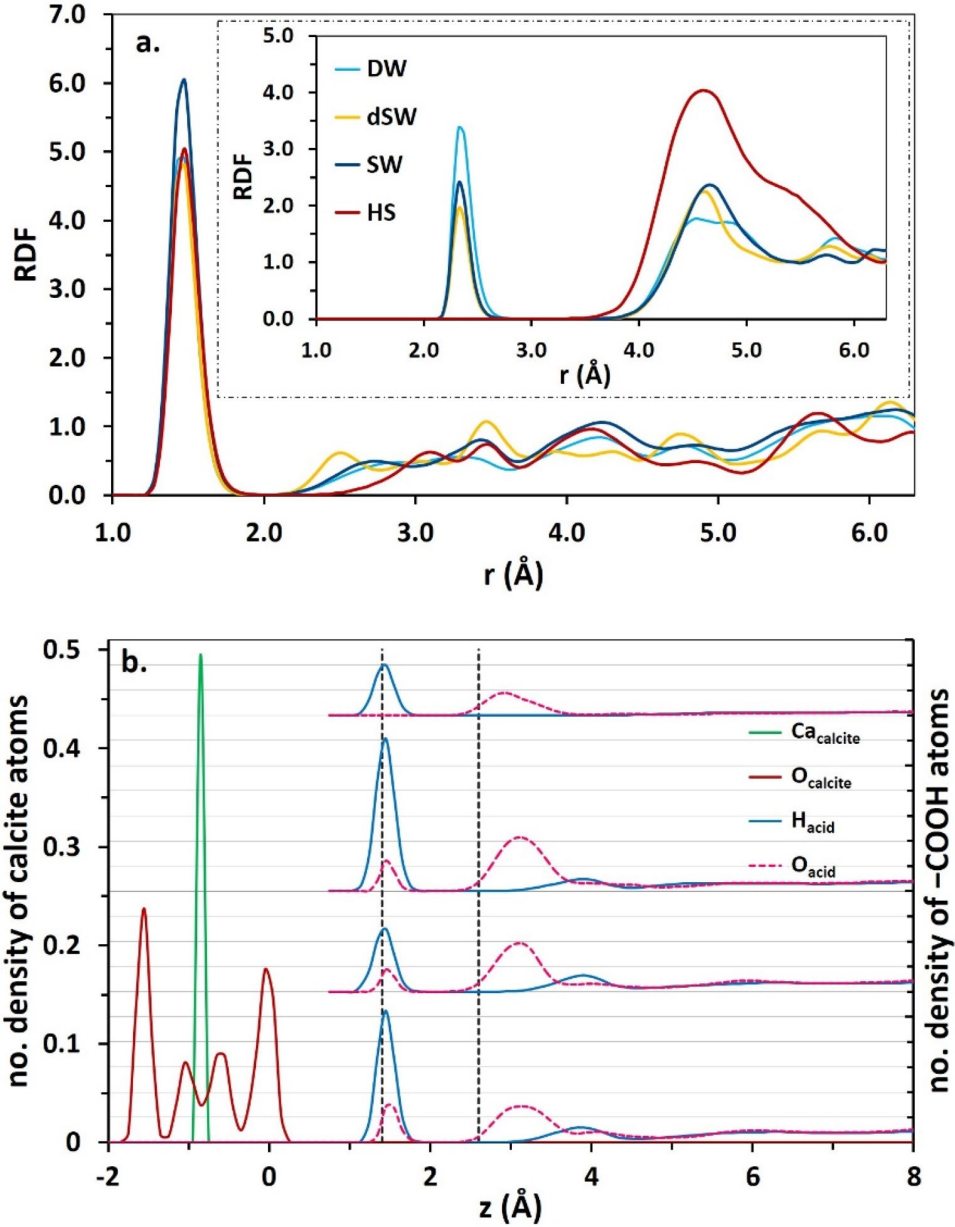
(**a**) RDF of O_calcite_-H_acid_ (main chart) and Ca-O_acid_ interactions (inset). (**b**) Density profiles of O_acid_ and H_acid_ (the sets in the right approximately beyond 1 Å) with respect to Ca and O_calcite_. Every set of –COOH densities respectively corresponds to DW, dSW, SW and HS from bottom to top. The gridlines are drawn to compare the scales of H_acid_ and O_acid_ densities at different salinities. The vertical dashed lines refer to the maxima positions of compact hydration layers over calcite.

Radial distribution function (RDF) is a convenient analysis for quantifying extent of interaction between two certain particles at micro-scale. RDF analysis reveals that Ca–O_acid_ and O_surface_–H_acid_ interactions advance BA adsorption onto calcite (Fig. 7a). The first shell positions of O_acid_ and H_acid_ around Ca and O_surface_, respectively at 2.33 Å and 1.48 Å, very satisfactorily match with the distances between these corresponding atoms calculated from their atomic density profiles shown in Fig. 7b. The density profiles of O_acid_ and H_acid_ at different salinities are shown in the right panel of Fig. 7b next to density profiles of Ca and O_calcite_, i.e., atoms comprising outermost layer of the calcite slab. Note that positions of the first and second hydration layers are shown as vertical dashed lines. The base level of number density profiles of O_acid_ and H_acid_ is set to zero in all four profiles in the right panel and the gridlines are drawn to compare their scales. The position at which H_acid_ density profile passes the maximum value, i.e., ≈1.5 Å, matches with the location of the first shell positions in H_acid_–O_calcite_, Fig. 7a. Meanwhile, the appearance of two maxima in the O_acid_ density profiles is in line with two shells of the O_acid_ atoms around the Ca, as verified by the inset of Fig. 7a. Density profiles show that BA diffuse into the water monolayer above calcite to directly adsorb onto the mineral via –COOH functional group. Twin modes of adsorption of BA onto calcite is also confirmed from simulation snapshots (see Fig. 3 and the inset of Fig. 4). In the first mode, the molecule develops hydrogen bonding with surface through the interaction of H_acid_ with O_calcite_. Geometric definition proposed by Luzar and Chandler^82^ was employed for determining hydrogen binding of BA molecules to basal carbonate groups. According to that criterion, two species develop H-bonding if their inter-oxygen distance is below 3.5 Å with a simultaneous H–O⋯O (here, H_acid_–O_acid_⋯O_calcite_) angle below 30°.

In the second mode, two simultaneous interactions are developed between BA and calcite. These interactions include an H-bonding, as in the first mode, together with Ca–O_acid_ interaction. These two modes of interaction are termed respectively as single mode and double mode throughout the text. The simulation snapshots indicate that BA needs to get closer to calcite in order to facilitate Ca–O_acid_ binding upon the double mode interaction (inset of Fig. 4). This is the reason behind the appearance of two density peaks of BA at calcite/water interface. However, in HS brine, only one peak is observed at the interface as a result of H-bonding in single mode interaction and double mode interaction is not observed and hence the Ca–O_acid_ RDF peak at 2.33 Å is also disappeared in the inset of Fig. 7a. The single mode interaction denotes the status when binding of the organic molecule to the surface is a result of single interaction between H_acid_ and O_calcite_. Meanwhile, in the double mode interaction, two simultaneous interactions are responsible for the binding of acid to calcite. These interactions include the formation of H-bonding, similar to the single mode, and an additional interaction between Ca atoms of the surface in the outermost layer and O_acid_.

Overall, increasing solution salinity weakens tendency (or likelihood) of BA molecules for pairing with basal carbonate groups (CO_3_^2−^) on the calcite surface, which is clearly reflected in the BA–O_calcite_ RDF plot (Figure S11, Supplementary Information). Indeed, extension of BAs adsorption is a function of all pairwise interactions between BA and calcite, BA and salt and salt and calcite. Figure S13 exhibits weakening interplay of BAs and Na^+^ cations at higher salinity levels because of the stronger association (pairing) of ions (Na^+^ and Cl^-^). Development of ionic cluster diminishes the number of available (free) ions for interacting with BA molecules. This in turn enhances the chance of self-hydrophobic pairing of BA compounds.

### Vertical (z-component) trajectory of BA molecules

**Figure 8:**
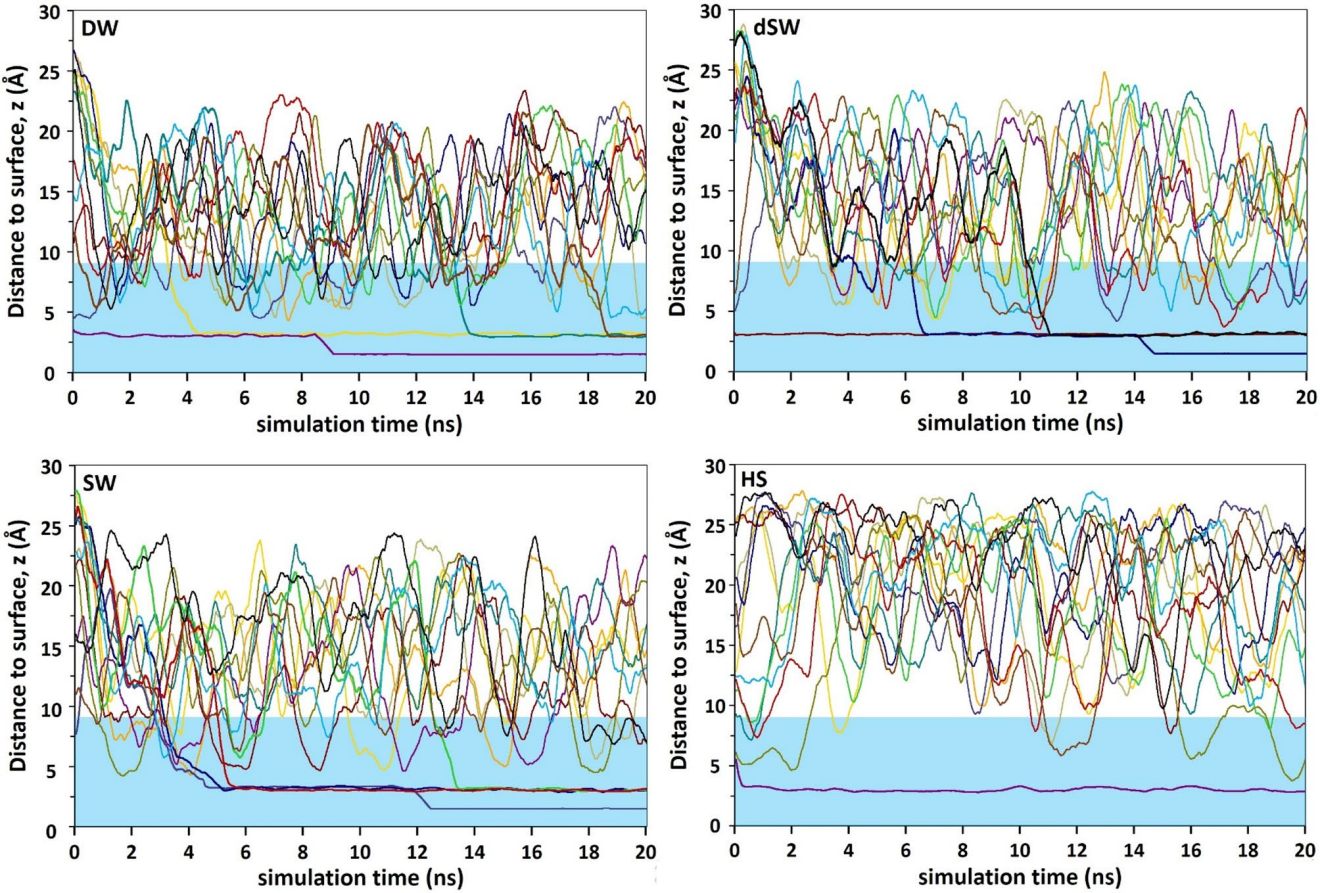
Vertical trajectories of BA over calcite surface at different salinities as a function of simulation time. Different BA molecules are traced by different colors. The shaded blue region corresponds to hydration layers over calcite (i.e. the ca. 1-nm thick interface).

In order to gain insight into likelihood of BAs for visiting calcite/water interface, we shall present z-component trajectory of those molecules over time, Fig. 8. MD simulations reveal that the affinity of BA to stick to calcite is very different in HS. Figure 8 shows the vertical trajectories of BA molecules as a function of simulation time above [1014] calcite plane for different salinities. Despite HS, in DW, dSW and SW brines, BA molecules can diffuse into the compact hydration layers covering calcite and strongly stick there for a prolonged time. Those acid molecules which cannot diffuse within water monolayers commonly wander within the 1-nm thick interface above calcite. From trajectories, some of them may find chance to penetrate hydration layers to tightly adsorb onto surface during the simulation. Once they overcome the hindrance of hydration layers and reach the mineral, they firmly stick there until the end of the simulation. The gap between those prolonged adsorbed BA molecules is another evidence of two modes of adsorption. In contrast, in HS, BA molecules reveal much less affinity to calcite and rarely diffuse into the 1-nm thick interface. As a rare instance, a unique BA which diffuse into the proximity of the hydrated calcite within the early period of simulation forms a single mode adsorption. This reduced hydrocarbon affinity to calcite in high salinity brine is the result of *screening effect* caused by high population of ions in HS. The simulation snapshot in Fig. 3 confirms the screening effect of ions as a result of their high population near the surface which in turn impedes the appearance of BA within 1-nm thick interface. In line with this observation, theoretical investigation by Wang et al. revealed the weaker attachment of epoxy onto concrete (hydrated calcium silicate) substrates in saline environments than deionized or dried conditions^83^. They noticed the weakened H-bonding network between adsorbed epoxy molecules and solid surface in response to the intruding water molecules. This effect is intensified in the presence of dissolved NaCl, thus facilitating epoxy liberation from a concrete matrix.

As observed in Fig. 5 in HS brine, an extra EDL-like layer is also observed beyond the original EDL over calcite. This extra charged layer intensifies the aforementioned screening effect and BA molecules are more seriously repelled off the interface than other brines.

### Potential of mean force (PMF)

**Figure 9:**
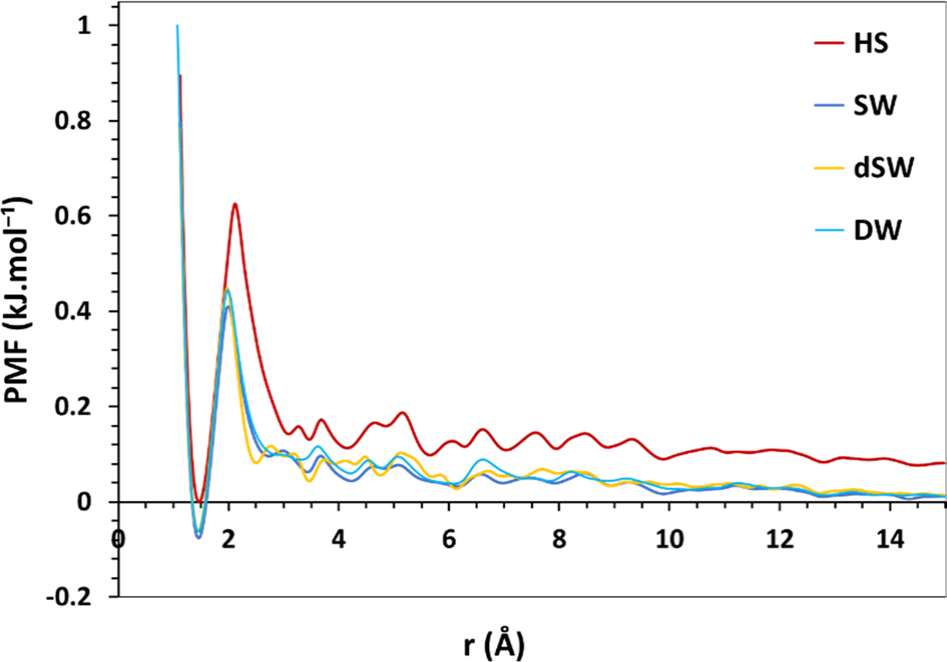
Potential of mean force curve for the adsorption of BA molecules onto calcite.

Potential of mean force (PMF) is shown in Fig. 9 as a function of separation between BA and calcite surface. PMF is defined as the average work needed to bring two species into contact at a distance r from an infinite separation. PMF is related to the radial distribution function between the two certain species by ${\text{PMF}} = - {\text{k}}_{{\text{B}}} {\text{T}}\ln {\text{g}}\left( {\text{r}} \right)$ where k_B_ is the Boltzmann constant and T is the temperature of the system in K. PMF is usually calculated to assess the effective pair interaction of two bodies in a simulation. Figure 9 shows the similar plot of PMF in different salinities. However, the condensation of ions in HS brine has a remarkable impact on the absolute value of free energy and leads to a higher energy barrier compared to brines with lower salinities. It is observed that there exists an energy barrier in all brines at ~ 1.5 Å with negative values of the free energy of BA adsorption onto calcite for DW, dSW and SW. Meanwhile, in HS brine, the adsorption free energy of the organic molecule onto the surface is almost zero. Here, we also observe that the energy barrier for the adsorption of BA onto calcite is 0.63 kJ/mol in HS brine while it ranges from 0.48–0.51 kJ/mol in the rest of salinities. The lowest value of free energy and the increased energy barrier of adsorption of BA onto calcite in HS brine stems from the screening effect of ions which inhibits adsorption of the organic molecules onto the mineral.

### 2D (Planar) distribution map

**Figure 10:**
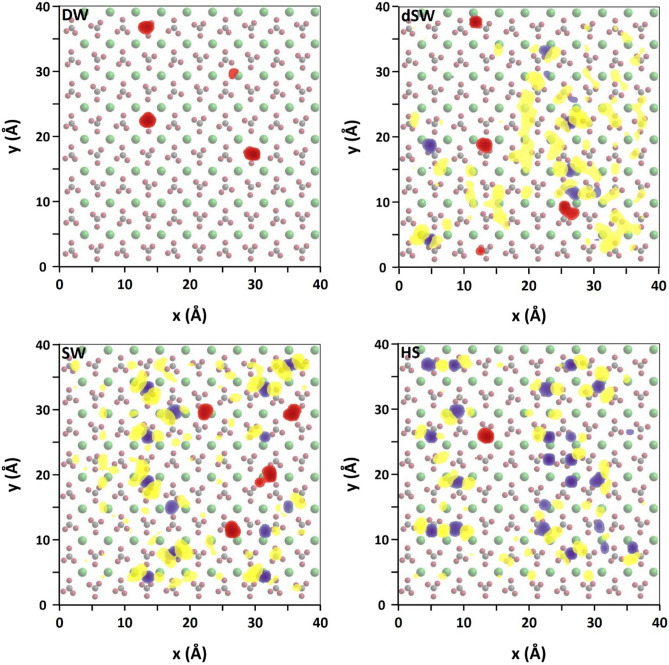
2D lateral density profile of Na^+^ (purple), Cl^−^ (yellow) and BA (red) at calcite/water interface at different salinities superimposed over the topmost calcite layer.

Thus far we figured out salinity-dependent propensity of BA and ions for loading nearby the calcite slab. However, the arrangement of those components over the calcite surface is not clear. Planar distribution map provides evidence for relating localization of ions and BAs to the structural features of the calcite surface and with respect to each other. Former analyses suggested that Na^+^ ions occupy oxygen sites of the surface and thus screen BA-calcite H-bonding. Cl^−^ ions which are triggered to get close to surface upon the attraction of sodium layer also impose screening effect against organics. Therefore, the formation of adsorbed salt layers over calcite reduces BA adsorption onto the surface. To validate these results, we performed statistically sound analysis to explore the average positioning of BA and ions over calcite surface. Thus a lateral 2D density map of Na^+^, Cl^−^ and BA was obtained within the compact hydration layers at calcite/water interface. The superposition of the lateral density maps on the topmost calcite layer is observed in Fig. 10. In all cases, BA firmly adsorb onto calcite over those sites not occupied by ions. Strong single/double mode adsorption is fairly observed by confined distribution of the molecules over the surface. Na^+^ ions also tightly lodge onto the mineral inferred by their dense purple marks. The maps reveal that Cl^−^ adsorption is mostly assisted by in situ Na^+^ ions. The density maps confirm that the dominant adsorption of ions over surface sites (mainly dangling O-atoms of carbonate groups) prevents BA adsorption onto calcite. The screening effect is much more critical in HS because the ions not only impose their hindrance directly over the mineral (as observed in lateral density maps) by occupying adsorption sites but also impede BA diffusion toward calcite beyond the interface as previously observed from simulation snapshot.

### Mean square displacement (MSD)

**Figure 11:**
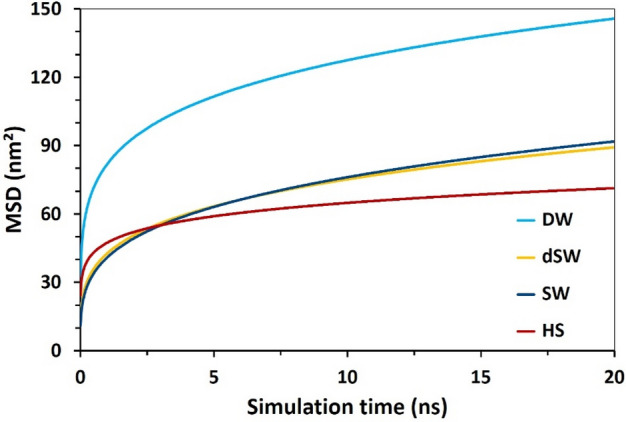
The mean square displacement of BA at different salinities.

**Figure 12:**
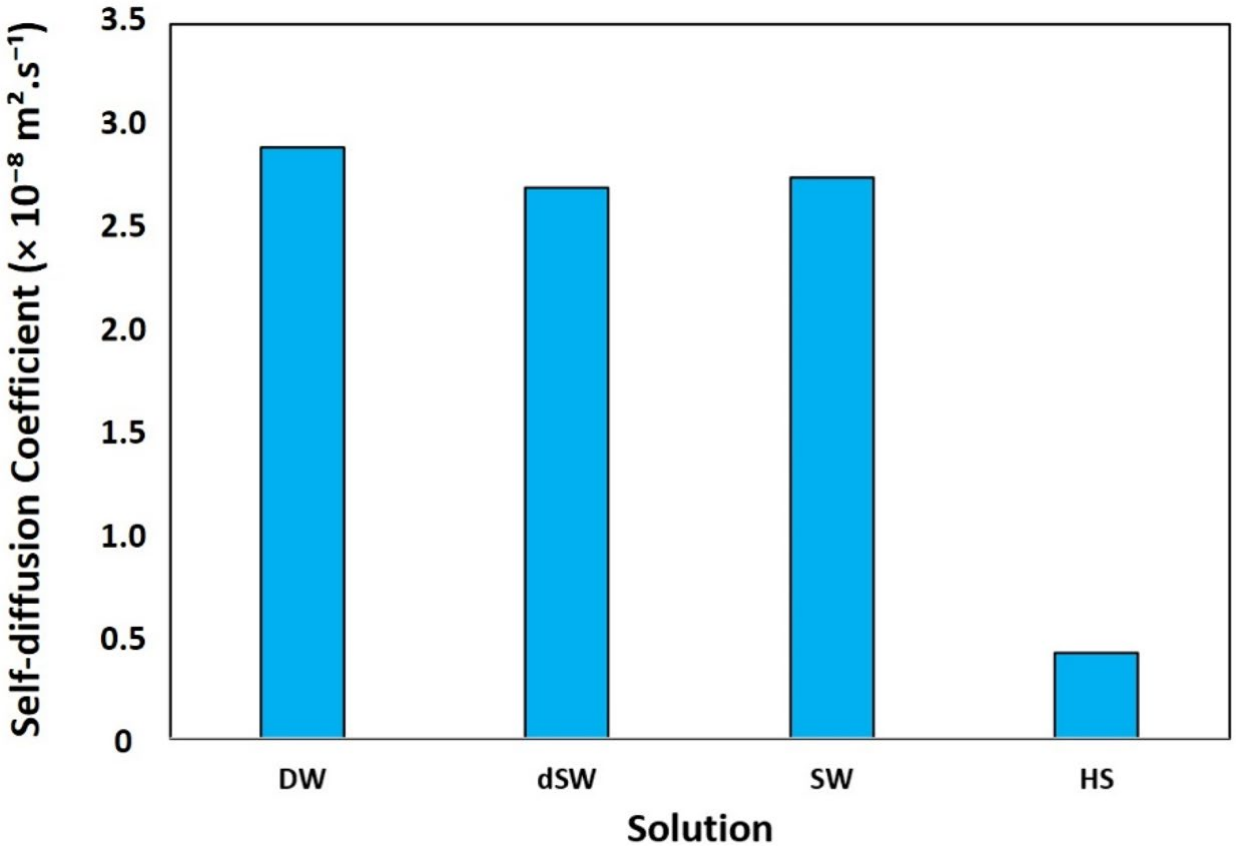
Self-diffusion coefficient of BA molecules in calcite/brine systems of different salinities.

MSD diagram, shown in Fig. 11, quantifies how far a certain compound explores the configurational space in the course of simulation defined as^84^:

1 $$MSD = \frac{1}{N}\left\langle {\mathop \sum \limits_{i = 1}^{N} \left| {\vec{r}_{i} \left( {t + t_{0} } \right) - \vec{r}_{i} \left( {t_{0} } \right)} \right|^{2} } \right\rangle_{{t_{0} }}$$

where summation goes over absolute displacement of total number (N) of BA molecules and the angular brackets denote ensemble average over reference timesteps t_0_. In precise terms, MSD is a statistical measure for the extent of spatial traverse of a particle with respect to a reference position over time^84^. This allows evaluating and comparing diffusive spreading of a species around the space. Self-diffusivity of BA was obtained (Fig. 12) for different saline solutions by processing atomic trajectories according to the popular Einstein’s relation^74^:

2 $$D = \mathop {\lim }\limits_{t \to \infty } \frac{MSD(t)}{{6t}}$$

Figure 11 displays the decreasing value of MSD at higher salinities, with the order: DW > dSW ≈ SW > HS. Furthermore, self-diffusivity coefficient (i.e., slope of MSD diagrams) of BA obeys the same trend (Fig. 12). The increasing trend of MSD for BAs in DW reflects high diffusive motion of those molecules in an ion-free medium. However, in HS, which contains a high population of ions, not only the MSD values moderately fall compared to other brines, but also the increasing trend of MSD clearly decelerates. The reduction of self-diffusivity of organic molecules in concentrated brine solutions has been pointed out in other investigations as well; for example, rapid decline of protein diffusivity at concentrated NaCl solutions^85^. This effect happens because of the larger volume occupied by ions at higher salinities limiting the available space for spreading of BA molecules, and in turn enhances the chance of those molecules for association as a result of hydrophobic (solvophobic) interaction and/or H-bonding of carboxylate groups. BA–BA RDF plot in Figure S12 confirms the stronger self-association of BA molecules at higher salinity level. Further, we notice in Figures S13–S14 the weakened tendency of BA compounds towards ions (Cl^−^ and Na^+^) at higher salinities. In sum, increasing salt concentration diminishes the dispersive movement of BAs in the electrolyte solution as a result of strengthened self-interaction and pairing of those molecules. Lastly we should emphasize that in all calcite/brine system, MSD curves transit to a linearly varying trend (i.e., constant slope) after initial 10 ns. Achieving a constant self-diffusivity coefficient indicates the sufficiency of the initial 10 ns for bringing the system to a micro-equilibrium state.

### Adsorption isotherm

**Figure 13:**
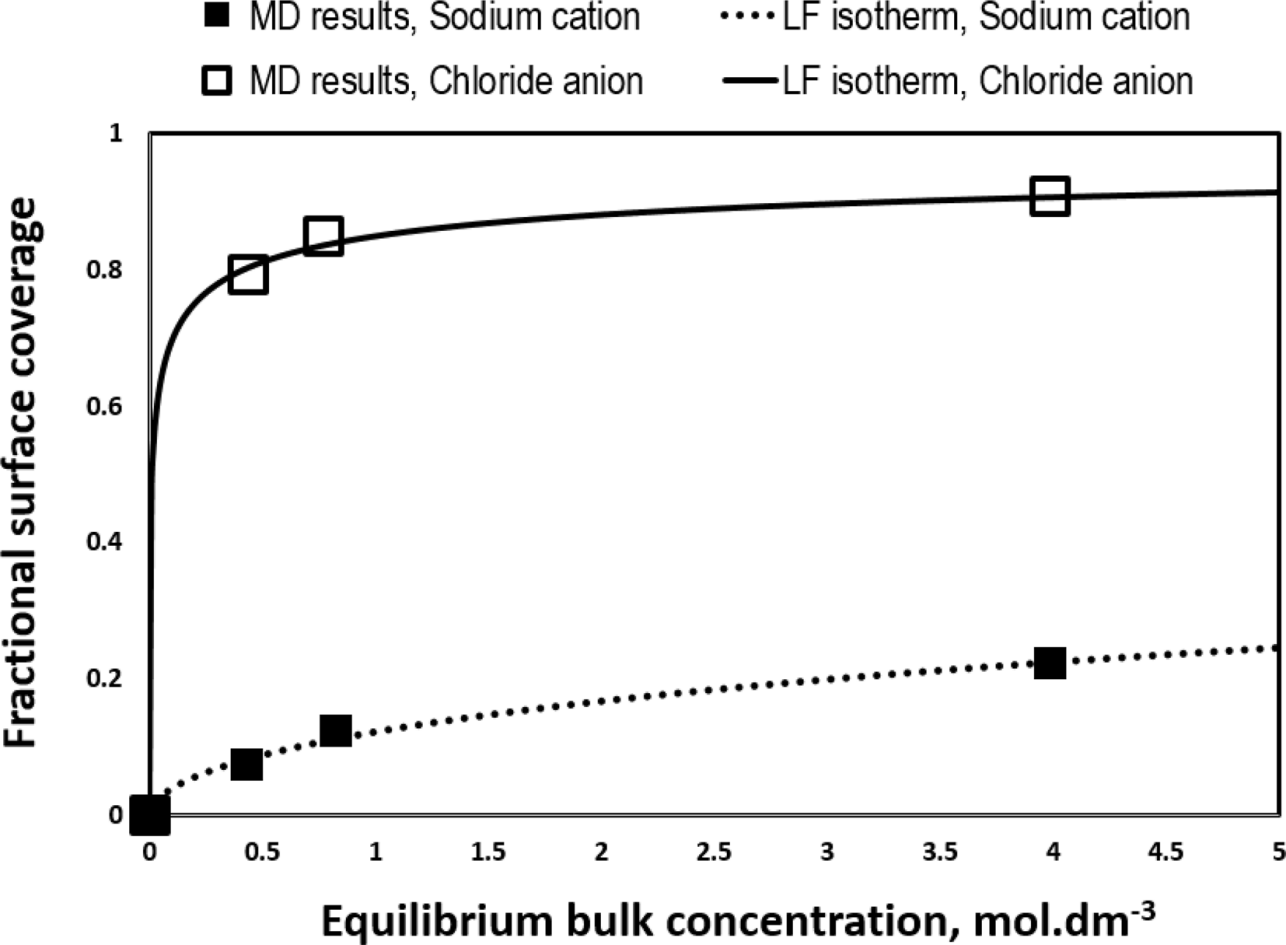
Adsorption isotherm of Na^+^ and Cl^−^ ions obtained by MD simulation (filled squares) together with the fitted FL isotherm.

Besides structural aspects presented above, here we assess the surface affinity of ions on the basis of the relation between adsorption intensity and bulk concentration, known as adsorption isotherm^78^. Figure 13 displays the fraction of the surface sites (i.e., protruding oxygen atoms of basal carbonate and surface residing Na^+^ cations for sodium and chloride ions, respectively) occupied versus equilibrium concentration within the bulk solution phase. Following a preliminary evaluation, we found the Freundlich–Langmuir (FL) isotherm best matching adsorption data, expressed as follow^86^:

3 $$\theta = \frac{{KC_{eq}^{n} }}{{1 + KC_{eq}^{n} }}$$

with $\theta$ and $C_{eq}$ denoting surface coverage ratio and equilibrium bulk concentration, respectively. The essence of interactions between adsorbent and adsorbate are incorporated to the model parameters K and n (≤ 1), with latter accounting for the deviation from a perfect Langmuir isotherm (i.e., n = 1).

As shown in Fig. 13, the fitted curve rapidly rises at low adsorbate concentrations, obeying a Freundlich-like isotherm. However, we notice an asymptotic behavior at concentrated condition, signifying Langmuir adsorption behavior. Therefore, the FL model should be considered as a generalized Langmuir isotherm when surface sites do not get fully occupied by adsorbates (herein Na^+^ or Cl^−^) at high concentrations. Adsorption data obtained by MD simulation were perfectly regressed by Eq. (1) with parameters K = 0.1386 and n = 0.5267 for Na^+^ and K = 5.656 and n = 0.392 for Cl^−^ (Fig. 13). Unlike the ideal Langmuir isotherm, fractional surface coverage shown in Fig. 13 tend to plateau at values much lower than complete occupation of the adsorbent sites, i.e., $\theta = 1$. This peculiarity basically arises by the intensified interaction of cation and anions in the bulk space upon concentrating the aqueous solution. As discussed earlier, increasing NaCl concentration enhances tendency of cations and anions for pairing and involvement in the ionic clusters.

Last but not least, It should be noted that in this paper, we have streamlined the very complex oil/brine/rock system into a simple model in which we ignored the role of potential determining ions (such as Mg^2+^, Ca^2+^, SO_4_^2−^, CO_3_^2−^ etc.) and only a likely mechanism among several possible ones has been studied. In our future research, the role of counter ions rather than NaCl to find a physical insight into the impact of potential determining ions for the implications to controlled/engineered salinity water flooding.

### Electronic charge density distribution

**Figure 14:**
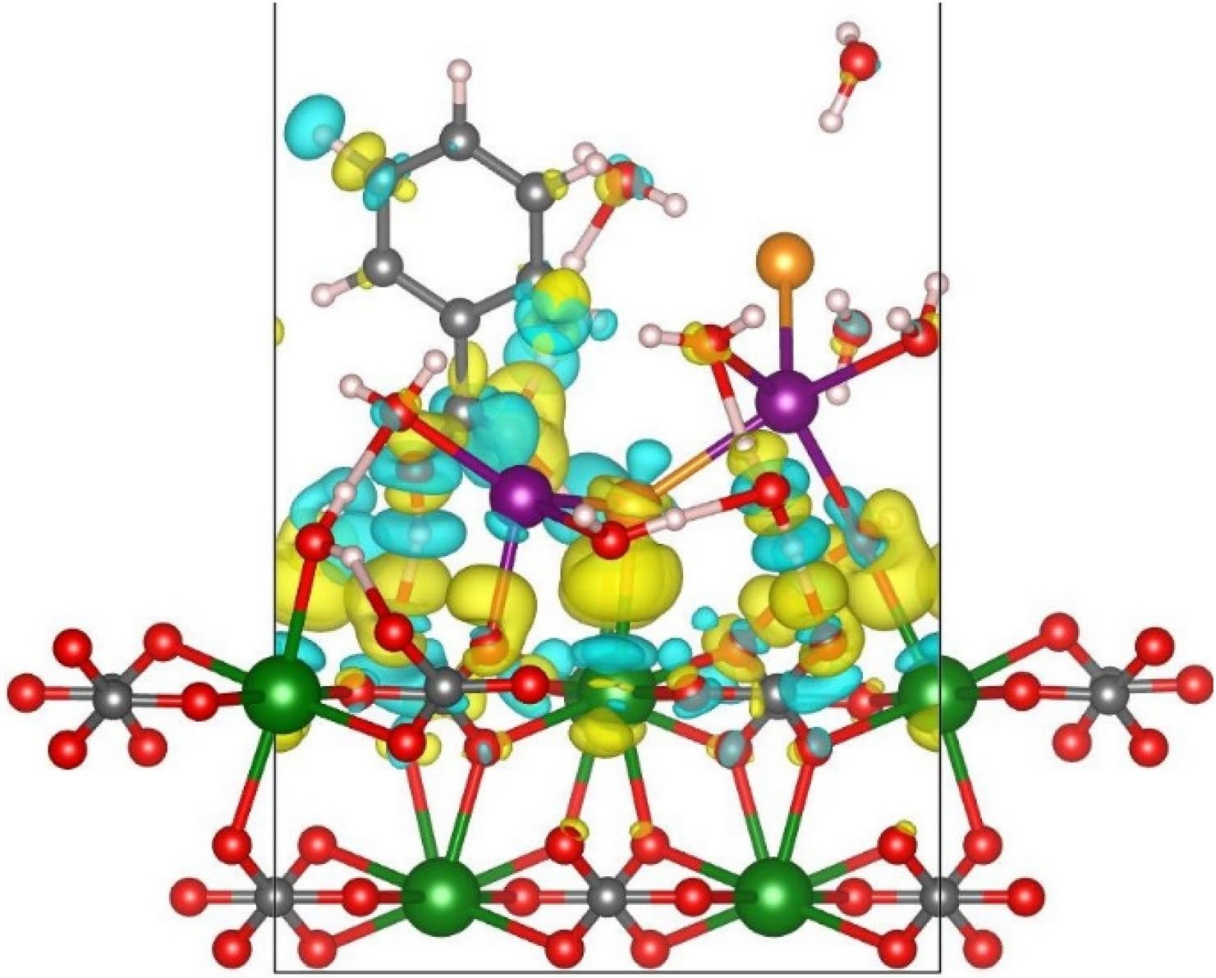
Charge density difference plot superimposed on optimized configurations. Electron accumulation and depletion are represented by yellow and blue areas, respectively. The atomic color codes are similar to those in other figures in the article except chlorides which are shown in orange to avoid confusion with charge density plots (see Supplementary Information for other pictures).

DFT results show that ions present in the brine induce charge redistribution (charge density difference) of the calcite surface due to the overlap of the orbitals of the ions and surface. Moreover, formation of H-bonding network in the proximity of the substrate is observed. In fact, the water molecules bind to the surface via the Ca–O_w_ interaction while H_water_ atoms point toward the protruding O_calcite_ above the surface to develop hydrogen bond. The H-bonding is facilitated by the herringbone arrangement of the topmost O_calcite_ atoms of carbonate groups in the [1014] calcite surface. The bonding charge density (charge density difference), ∆*ρ*, was calculated by means of the following equation where *ρ*_total_, *ρ*_calcite_ and *ρ*_ads_ are respectively the charge density of the whole system (including water, ion and BA adsorbed onto calcite), the bare surface and individual species in z-direction. In Fig. 14, the yellow areas and the blue ones represent accumulation and depletion of the electron charge, respectively. It is expected to observe the strengthening of the bond between atoms in the yellow region as a result of high density of electrons, while the bonds are weakened in the blue regions due to the electron deficiency. There exist areas of large charge accumulation between O atoms of adsorbed water molecules and Ca sites on the surface which is due to the electron transfer from Ca to O_water_. Electron transfer from Ca to water is validated by the electron deficiency at Ca atoms, as recognized by blue colors in Fig. 14. During water adsorption, there exist an obvious charge accumulation region between water molecules and O atoms of adjacent carbonate groups on the calcite surface which is the result of the formation of hydrogen bonding between H_water_ and O_calcite_. The appearance of a blue region on those H_water_ molecules which contribute in H-bonding validate the electronic charge depletion on the atoms due to charge transfer toward O_calcite_ during H-bond formation. Charge density difference verifies that the interfacial water structure originates from the H-bonding network between water molecules at the interface. The plot shows penetration of ions into H-bonding network at the interface where the accumulation of charge density between Na^+^—O_calcite_ and Cl^−^—Ca is observed in the Fig. 14. These interactions are expected to stabilize the network at the interface and are strong enough to extend to higher levels above the surface at subsequent layers. The charge density difference shows that BA can also contribute in this network by means of its –COOH functional group. The accumulated charge density between H_acid_ and O_calcite_ verifies the formation of H-bonding between BA and calcite. Meanwhile, O_acid_ creates H-bonding with adsorbed water molecule onto calcite to share in hydration layer over calcite. The charge density difference on BA shows that charge distribution is mostly expected around –COOH functional group which seems to be exclusively responsible for the interaction of the organic molecule with the surface and its contribution in H-bonding network above the surface.

## Conclusions

In this research, we performed molecular dynamics simulations to explore the affinity of a polar aromatic hydrocarbon (mimicked by benzoic acid, BA) to preferentially adsorb onto calcite mineral in NaCl brines of different salinities. Despite a persisting water monolayer, BA directly adsorbs onto calcite via its –COOH functional group by developing hydrogen bonding with O-atoms of surface carbonate groups. The preferential adsorption of Na^+^ over dangling O-atoms of surface which also triggers the attraction of Cl^−^ towards the surface forms salt layers at the interface which limits BA adsorption onto calcite. This trend is intensified in high salinity brine in which the accumulation of ions above the surface drastically reduces BA-calcite interaction. Indeed, high population of ions screens the development of hydrogen bonding between BA and calcite. This screening effect caused by ions at calcite/brine interface retards BA migration towards the mineral and as a result lowers the affinity of the molecule to adsorb onto the surface.

Our findings in this study has clear implications for low salinity waterflooding  as a means for oil recovery. According to the results, dilution of a formation brine containing purely Na^+^/Cl^-^ions, would not result in further detachment of polar oil (BA) groups, rather a wettability reversal toward a more-oil-wetting would be observed. Therefore, the maximal oil recovery must be attained at an optimal concentration of injection brine modified by e.g. potential determining ions such as Mg^2+^, Ca^2+^, SO_4_^2−^, CO_3_^2−^ etc.

## Supplementary Information

Supplementary Information.
