# Supplementary material for: Atomistic insight into salinity dependent preferential binding of polar aromatics to calcite/brine interface: implications to low salinity waterflooding
Source: Sci Rep. 2021 Jun 7;11:11967. doi: 10.1038/s41598-021-91402-0 (PMC8184864; doi:10.1038/s41598-021-91402-0)
Supplement: Supplementary file 1 — Supplementary Information. [file 41598_2021_91402_MOESM1_ESM.pdf]

Supporting Information

Atomistic Insight into Salinity Dependent Preferential Binding of Polar Aromatics to Calcite/Brine Interface: Implications to Low Salinity Waterflooding

Mohammad Mehdi Koleini*, Mohammad Hasan Badizad†, Hassan Mahani, Ali Mirzaalian Dastjerdi, Shahab Ayatollahi‡, Mohammad Hossein Ghazanfari

[1] Department of Chemical and Petroleum Engineering, Sharif University of Technology, Tehran, Iran.
* Corresponding Author; **Email:** mmkoleini@che.sharif.edu & mmkoleini@gmail.com
† **Email:** mohammadhasan.badizad@che.sharif.edu
‡ **Email:** shahab@sharif.edu

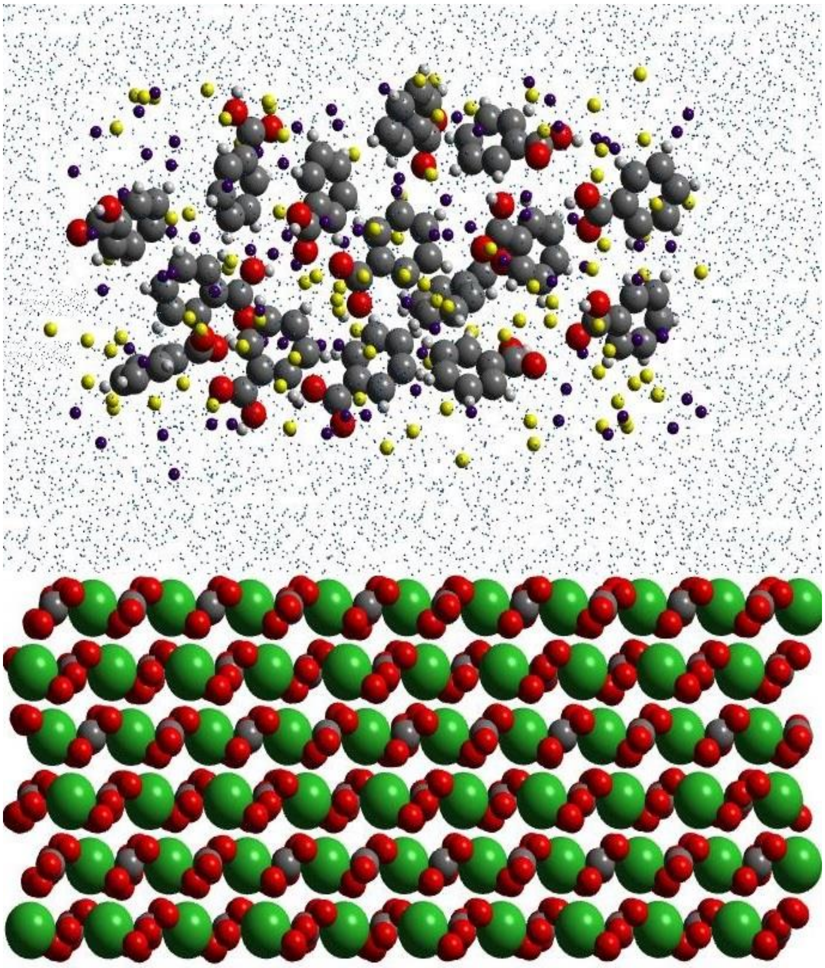

Figure S1 Initial configuration of a typical simulation ensemble adopted in this study: 15 BA molecules and sufficient number of ions required to satisfy the desired concentration of the brine were randomly inserted into the middle of the ensemble, respectively. These species were inserted at locations at least 10 Å above the outermost layer of the substrate to avoid any coercive interaction of components with surface due to close proximity. Afterwards, the simulation ensemble was filled with 1350 water molecules. Note that the initial coordinates of BA molecules are identical in all simulations to avoid the effect of their initial position on the final adsorption structure onto calcite. Also, the number of NaCl pairs are different in brines as follows: 0 in DW, 13 in dSW, 26 in SW and 110 in HS. This snapshot is taken from the calcite/brine system with highest salinity (HS) at the beginning of simulation. For clarity, we reduced the size of ions (illustrated by purple and yellow spheres representing sodium and chloride, respectively) and water species (shown as blue dots).

Table S1 forcefield parameters used for MD simulation

Atomwise LJ parameters and partial atomic charges for $CaCO_3$

Atom type	Partial charge	$\epsilon\ (kcal.mol^{-1})$	σ (Å)
$Ca_{calcite}$	+1.668	0.478	2.370
$O_{calcite}$	+0.999	0.139	3.090
$C_{calcite}$	-0.889	0.088	3.823

Atomwise LJ parameters and partial atomic charges for water (SPC/E model)

O_w	-0.834	0.1521	3.1507
H_w	+0.417	0.0000	0.0000

Interatomic LJ parameters for $CaCO_3$ and water

$C_{calcite}$	$O_{calcite}$	6.270	1.197
$O_{calcite}$	$O_{calcite}$	0.000273	4.744
$Ca_{calcite}$	O_w	0.270056	2.760
$C_{calcite}$	O_w	0.116185	3.469
$O_{calcite}$	O_w	0.146128	3.118
$O_{calcite}$	H_w	6.97252e-6	4.497

Atomwise LJ parameters for ions

Na^+	+1.000	0.0028	3.33
Cl^-	-1.000	0.1178	4.42

Atomwise LJ parameters and partial atomic charges for benzoic acid[1]

C_{phenyl}	-0.1150	0.070	3.550
H_{phenyl}	+0.1150	0.030	2.420
$C_{phenyl,\ carboxylic}$	-0.1150	0.070	3.550
$C_{carboxylic}$	+0.6350	0.105	3.750
$OH_{carboxylic}$	-0.5300	0.170	3.000
$O_{carboxylic}$	-0.4400	0.210	2.960
$HO_{carboxylic}$	+0.4500	0.000	0.000

Bond stretching parameters[2]

Bond type		Stiffness, $K_b\ (kcal.\text{Å}^{-2}mol^{-1})$	Bond length, (Å)
$C_{calcite}$	$O_{calcite}$	314.5	1.294
O_w	H_w	450.0	0.9572
C_{phenyl}	H_{phenyl}	367.0	1.080
C_{phenyl}	C_{phenyl}	469.0	1.400
C_{phenyl}	$C_{phenyl,\ carboxylic}$	469.0	1.400
$C_{phenyl,\ carboxylic}$	$C_{carboxylic}$	469.0	1.409
$C_{carboxylic}$	$O_{carboxylic}$	656.0	1.250
$C_{carboxylic}$	$OH_{carboxylic}$	450.0	1.364
$OH_{carboxylic}$	$HO_{carboxylic}$	553.0	0.960

Angle bending parameters[3]

Angle type			Stiffness, $K_b\ (kcal.deg^{-2}mol^{-1})$	Angle, (deg)
C_{phenyl}	$C_{phenyl,\ carboxylic}$	$C_{carboxylic}$	63.0	120.0
$C_{carboxylic}$	$OH_{carboxylic}$	$HO_{carboxylic}$	35.0	113.0
H_{phenyl}	C_{phenyl}	C_{phenyl}	35.0	120.0
H_{phenyl}	C_{phenyl}	$C_{phenyl,\ carboxylic}$	35.0	120.0
H_w	O_w	H_w	55.0	104.52
C_{phenyl}	C_{phenyl}	C_{phenyl}	63.0	120.0
C_{phenyl}	C_{phenyl}	$C_{phenyl,\ carboxylic}$	63.0	120.0
$C_{phenyl,\ carboxylic}$	$C_{carboxylic}$	$O_{carboxylic}$	80.0	120.4
$C_{phenyl,\ carboxylic}$	$C_{carboxylic}$	$OH_{carboxylic}$	70.0	120.0
$O_{carboxylic}$	$C_{carboxylic}$	$OH_{carboxylic}$	80.0	121.0
$O_{calcite}$	$C_{calcite}$	$O_{calcite}$	221.3	120.0

Dihedral torsion parameters[4]

Dihedral type				K_1	K_2	K_3	K_4
H_{phenyl}	C_{phenyl}	C_{phenyl}	H_{phenyl}	0.0	7.25	0.0	0.0
H_{phenyl}	C_{phenyl}	C_{phenyl}	C_{phenyl}	0.0	7.25	0.0	0.0
H_{phenyl}	C_{phenyl}	C_{phenyl}	$C_{phenyl,\ carboxylic}$	0.0	7.25	0.0	0.0
H_{phenyl}	C_{phenyl}	$C_{phenyl,\ carboxylic}$	$C_{carboxylic}$	0.0	7.25	0.0	0.0
H_{phenyl}	C_{phenyl}	$C_{phenyl,\ carboxylic}$	C_{phenyl}	0.0	7.25	0.0	0.0
C_{phenyl}	C_{phenyl}	C_{phenyl}	C_{phenyl}	0.0	7.25	0.0	0.0
C_{phenyl}	C_{phenyl}	C_{phenyl}	$C_{phenyl,\ carboxylic}$	0.0	7.25	0.0	0.0
C_{phenyl}	C_{phenyl}	$C_{phenyl,\ carboxylic}$	$C_{carboxylic}$	0.0	7.25	0.0	0.0
C_{phenyl}	C_{phenyl}	$C_{phenyl,\ carboxylic}$	C_{phenyl}	0.0	7.25	0.0	0.0
$C_{phenyl,\ carboxylic}$	$C_{carboxylic}$	$OH_{carboxylic}$	$HO_{carboxylic}$	4.0	5.00	0.0	0.0
$O_{carboxylic}$	$C_{carboxylic}$	$OH_{carboxylic}$	$HO_{carboxylic}$	0.0	5.00	0.0	0.0
$OH_{carboxylic}$	$C_{carboxylic}$	$C_{phenyl,\ carboxylic}$	C_{phenyl}	0.0	5.00	0.0	0.0

[1] Atom types for a benzoic molecules are defined in the sketch below.

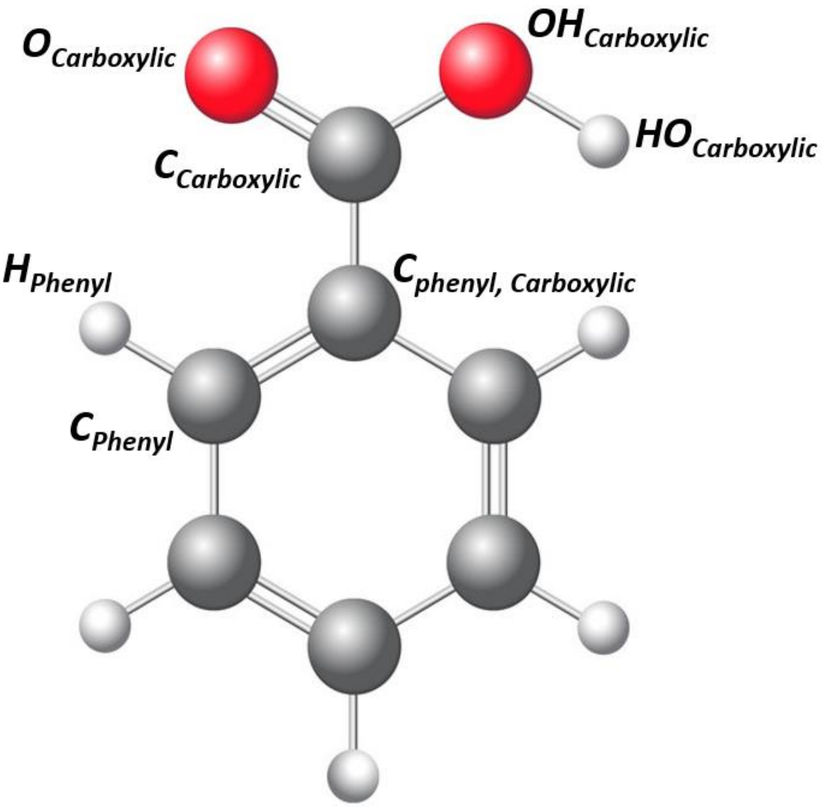

[2] Bonding parameters are formulized by: $E_{bond} = \sum_i K_i^r \left(r_i - r_{i0}\right)^2$

[3] Angle parameters are described as: $E_{angle} = \sum_i K_i^\theta \left(\theta_i - \theta_{i0}\right)^2$

[4] Dihedral parameters are defined as: $E_{dihedral} = \frac{1}{2}\sum_i \sum_{n=1}^{4} K_{n.i}^{\varphi}\left[1 +\right.$

In the above equations r_0 and θ_0 are equilibrium bond lengths and angles, with corresponding stiffnesses of K^r and K^θ. ϕ_i and K_n^φ denote dihedral angle and stiffness constants, respectively.

S1. Evaluating finite size effect

Simulation system was designed large enough to preclude any potential artefact due to finite-size effect. For this purpose, a complementary simulation was performed for a larger calcite/SW (60,000 ppm) system with box dimensions 56.67 Å x 54.89 Å x 19.77 Å for a longer timespan, 50 ns. By doing so, we acquired number and charge density distribution profiles (**Figures S2 and S3**) identical to **Figures 2** and **5**. Also note the similarity of MSD diagram (**Figure S4**) to that presented in **Figure 11**. This comparison confirms size-independency of our MD results.

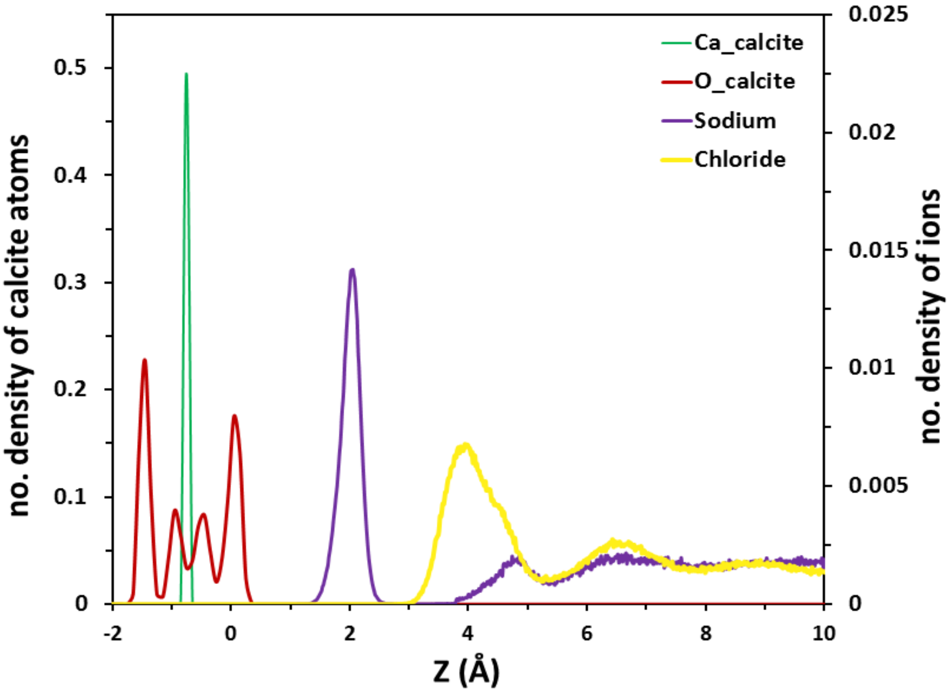

Figure S2 Density distribution diagrams for the larger calcite/SW system.

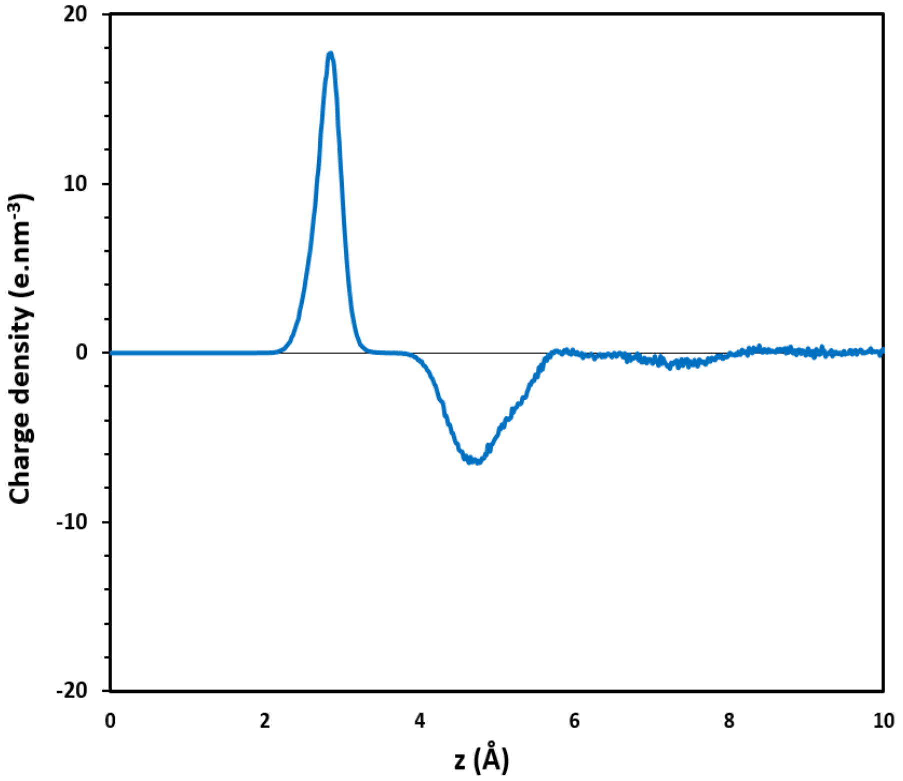

Figure S3 Charge density distribution for the larger calcite/SW system.

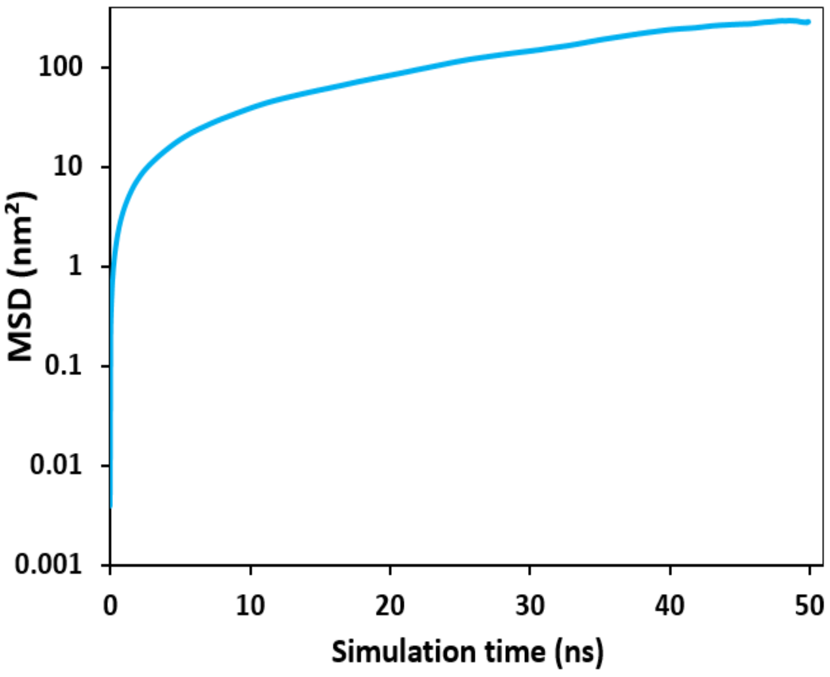

Figure S4 Time varying MSD diagram of BA molecules for the larger calcite/SW system.

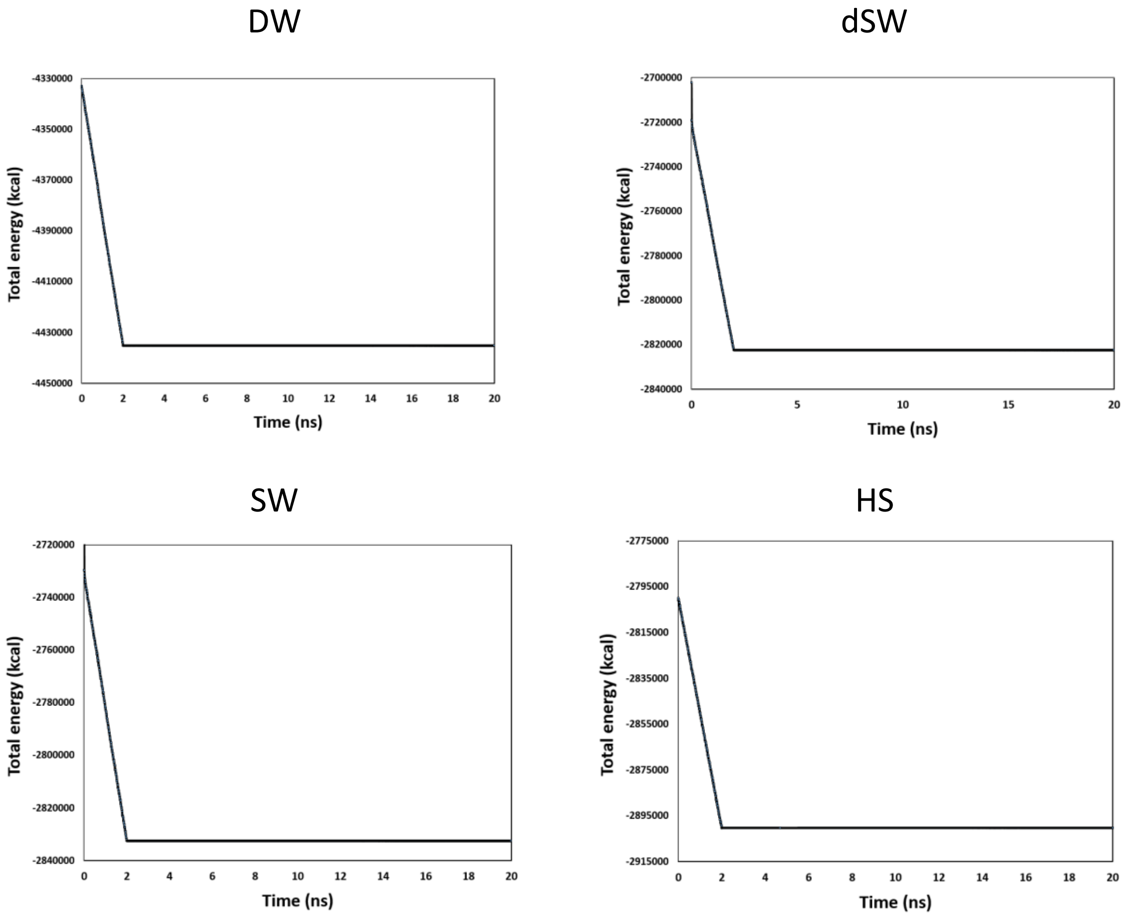

Figure S5 Time-variation of the total energy for different calcite/brine systems.

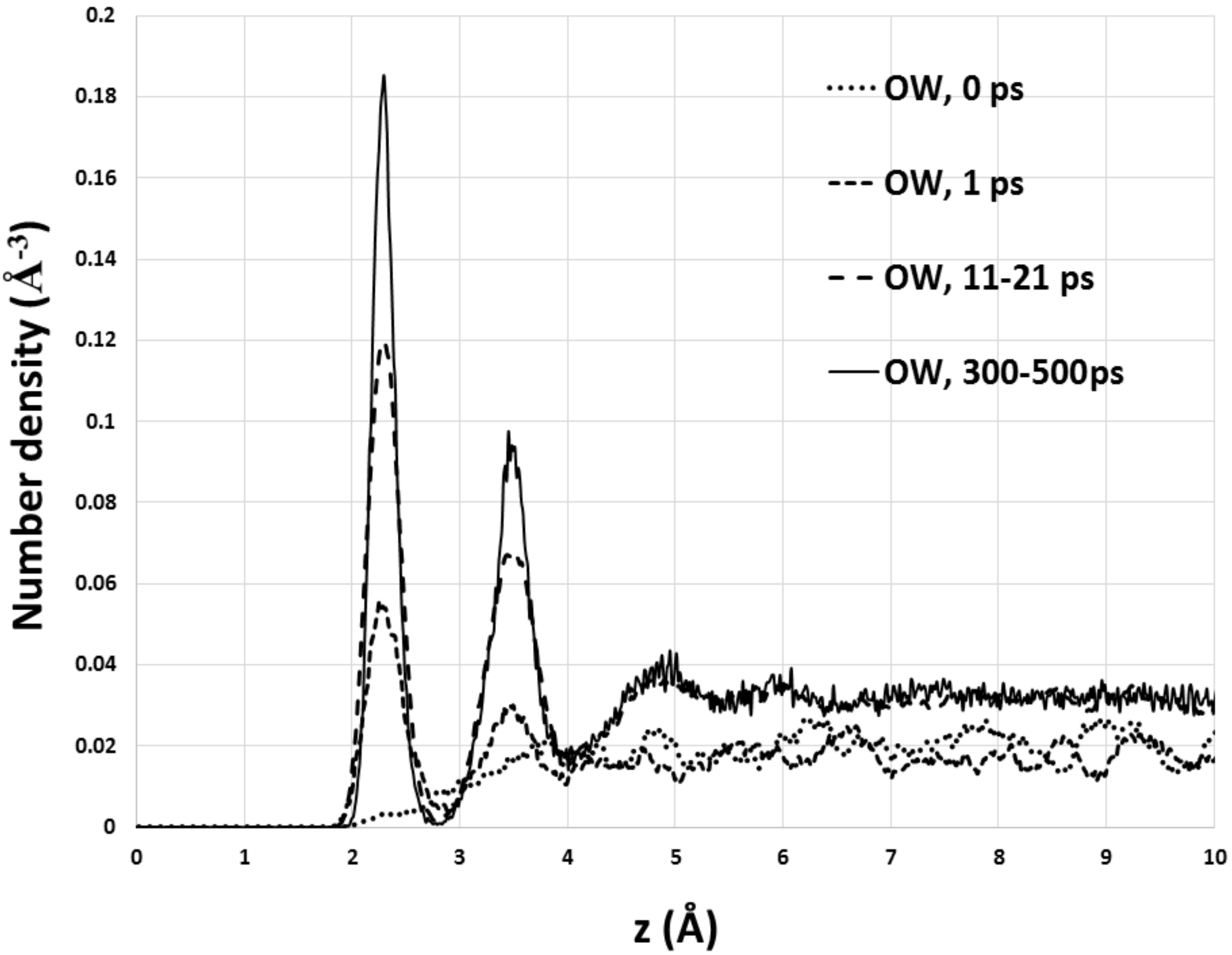

Figure S6 Development of wetting layers over the calcite surface over simulation time.

S2. Molecular orientation

Here, we present the calculation procedure for obtaining the angle distribution map (**Figure 6**). First, an axis is defined for each BA molecule (**Figure S7**) passing through the centroid of the BA phenyl ring and the carboxylic group. This axis is defined for each BA molecules as an vector, namely, $\vec{a} = (a_{x,}, a_y, a_z)$.

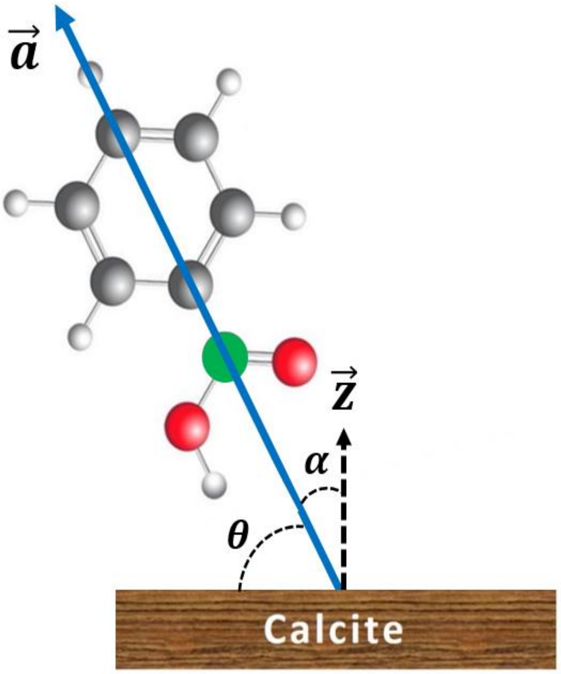

Figure S7 The schematic illustration of the inclination angle, θ, between benzoic acid molecules and calcite surface.

As illustrated in **Figure S7**, the angle ($\boldsymbol{\alpha}$) between that vector and normal to the calcite surface, *i.e.*, $\vec{z} = (0,0,1)$, is calculated using the inner (dot) product operation as follows:

$$cos\boldsymbol{\alpha} = \frac{\vec{a}.\vec{z}}{|\vec{a}||z|} = \frac{(a_x,\ a_y,\ a_z).(0,\ 0,\ 1)}{\sqrt{a_x{}^2 + a_y{}^2 + a_z{}^2}} = \frac{a_z}{\sqrt{a_x{}^2 + a_y{}^2 + a_z{}^2}} \qquad \text{(Eq. S1)}$$

and the desired inclination angle is readily obtained by:

$$\boldsymbol{\theta} = 90 - \boldsymbol{\alpha} \qquad \text{(Eq. S2)}$$

The preceding calculation was performed for each BA molecule at a series atomic trajectories produced every 2.5 ps. At each timeframe, a set of conceptual bins (**Figure S8**) is assumed parallel to the calcite surface and the angle calculated for each molecules is assigned to the location (z-component) of the corresponding BA molecule in a bin. Spatial position of a BA molecules is defined by the z-component coordinate of the carbon atom in the carboxylic group, as highlighted by green filled circle in **Figure S7** above. By performing this calculation within the space 1 nm apart from the calcite surface and a range of angle values [0, 90] we come up with a 2D histogram which was shown as a normalized distribution map in **Figure 6** of the main text.

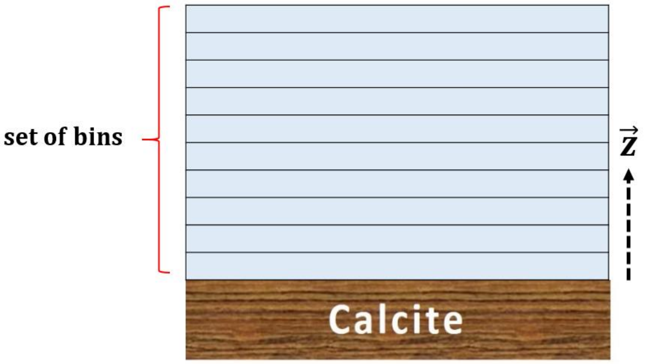

Figure S8 Sketch of the conceptual bins assumed for tracking z-component spatial position of each BA molecule.

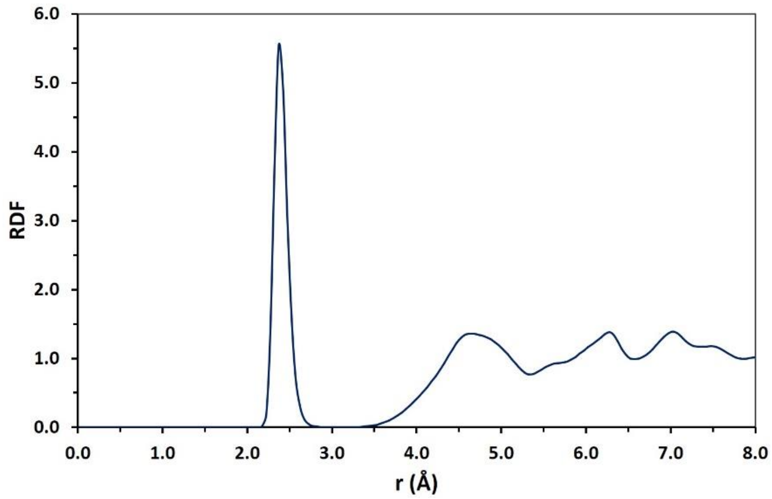

Figure S9 Ca-O_w RDF.

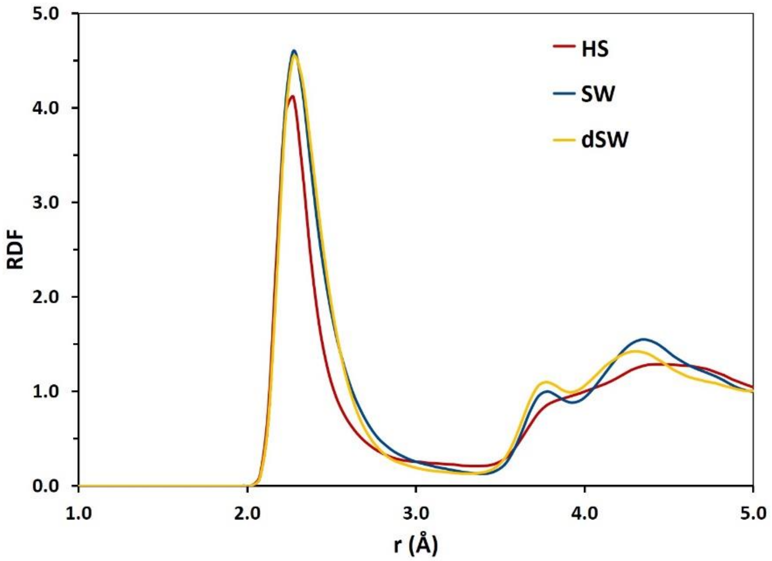

Figure S10 Na^+-$O_{calcite}$ RDF at different solutions.

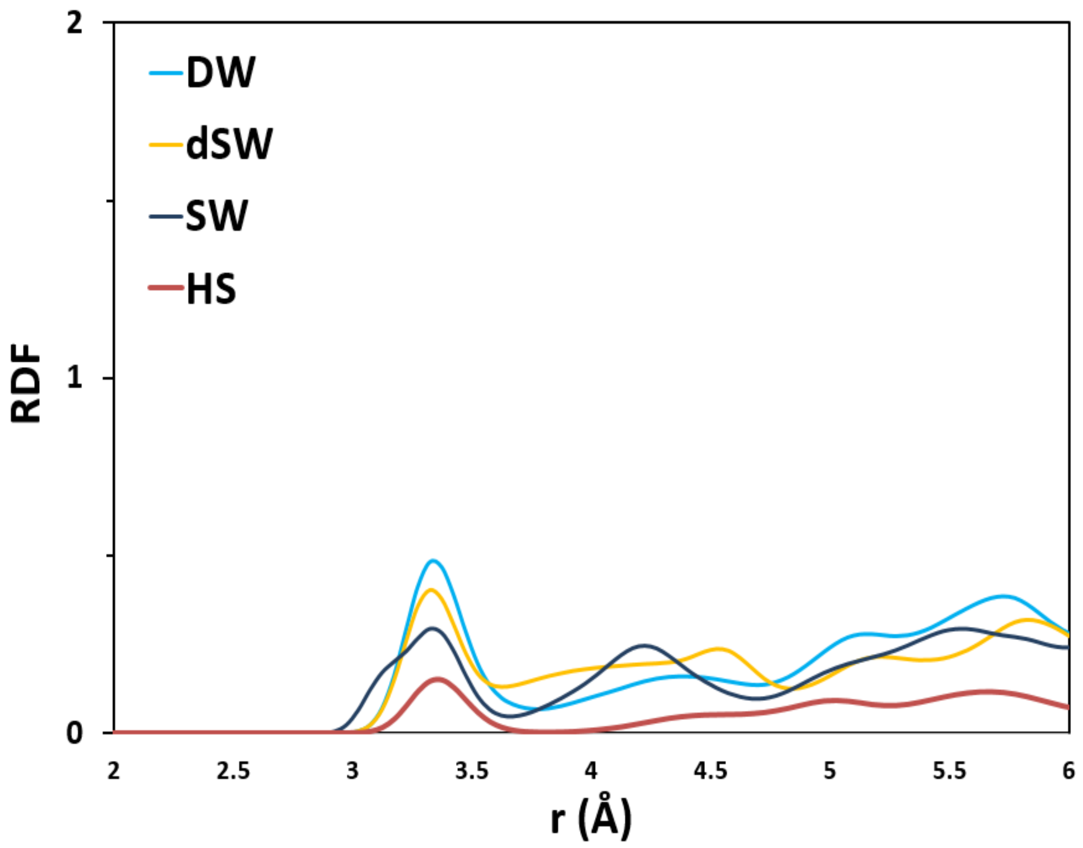

Figure S11 RDF of BA-O$_{calcite}$ at different salinity levels.

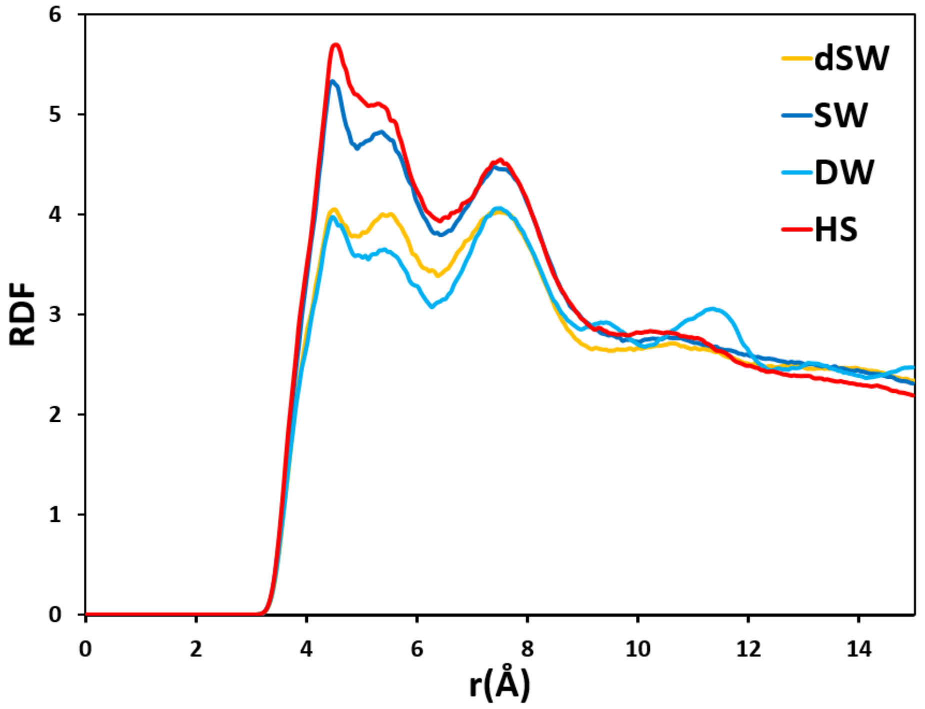

Figure S12 BA-BA RDF profile for solutions of varying salinities.

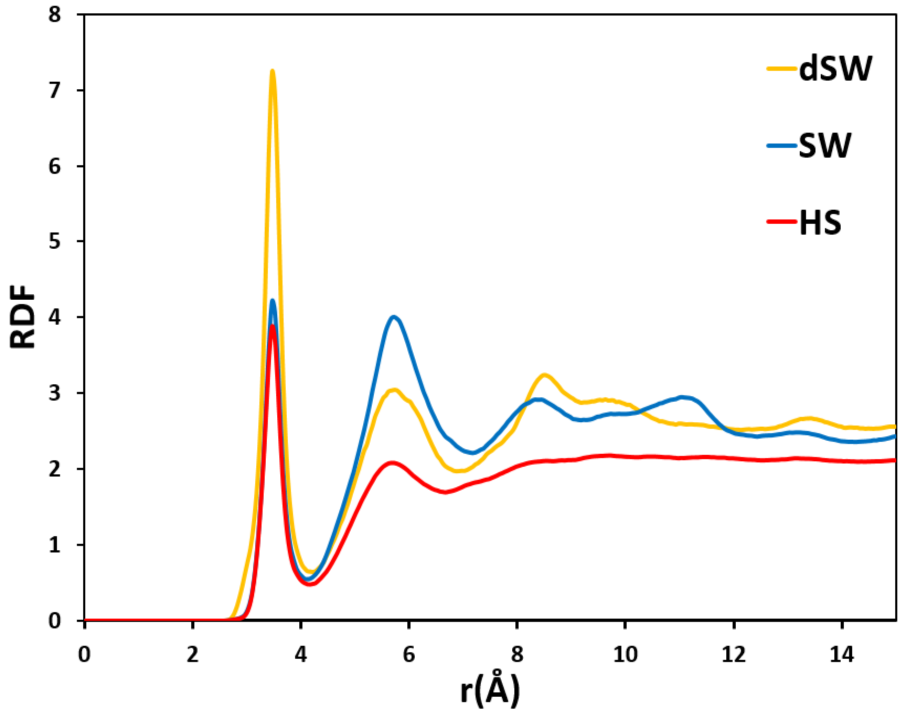

Figure S13 BA-Na^{+} RDF profile for solutions of varying salinities.

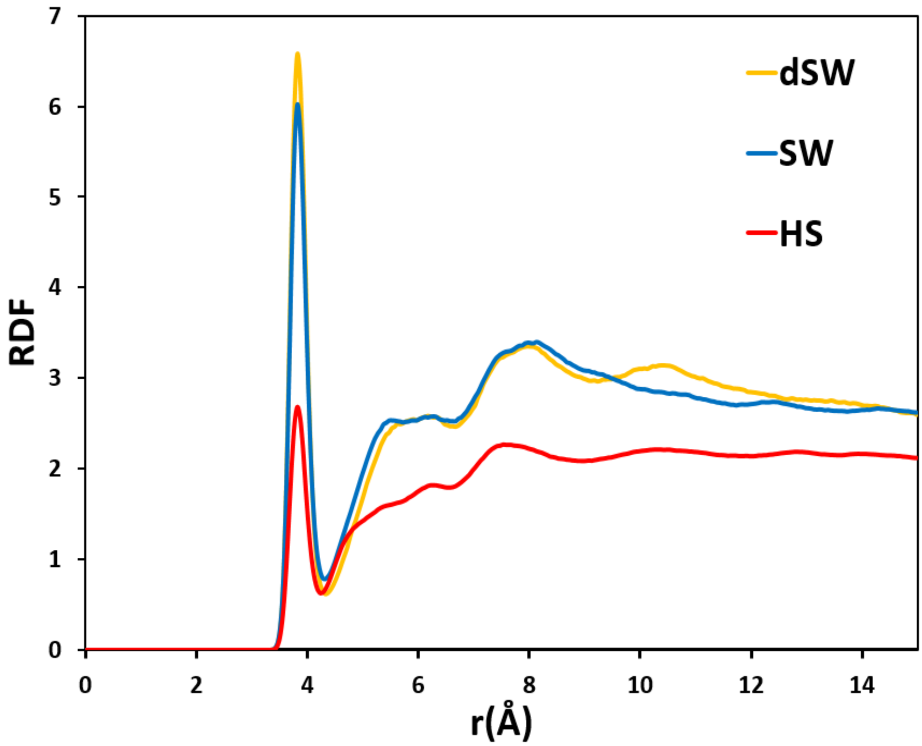

Figure S14 BA-Cl^- RDF profile for solutions of varying salinities.

S3. Details of DFT calculations

Quantum calculations were performed by means of a Plane-Wave Self-Consistent Field (PWSCF) method based on Density Functional Theory (DFT) as implemented in the Quantum Espresso software package. Long-range dispersion forces were included in our calculations with the aid of the semi-empirical Grimme DFT-D2 correction term which is crucial for an accurate description of calcite interlayer interactions and adsorption phenomena. We used the generalized gradient approximation (GGA) with the Pedrew-Burke-Ernzerhof (PBE) functional to account for the electronic exchange-correlation potential. Ultrasoft pseudopotential was employed to define the core electrons by plane-wave functions which have been taken from the Quantum Espresso pseudopotential library. The kinetic energy for wave functions and charge density were truncated at a cutoff of 50.0 and 500.0 Ry, respectively. We used a (2 × 2) surface unit cell of $(10\bar{1}4)$ calcite cleavage plane with two layers to study the adsorption of water, ions and BA. Twelve water molecules and 2 ion pairs of Na^+/Cl^- were placed on a calcite supercell with dimensions of 8.095×9.98×18.42 Å^3. The simulations were performed by adopting periodic boundary conditions as if they occur in an infinite space. To avoid interactions between periodic slabs, a vacuum region of at least 10 Å was placed above the water phase. The geometry optimizations were performed to find the optimum adsorption sites of adsorbates on calcite.

To this end, all atoms were allowed to relax until the magnitude of the residual force on all atoms reached 0.02 ev/Å and the total energy converged to 0.0001 eV. Integration over the Brillouin zone was found to fulfill convergence criteria with a (2 × 2 × 1) k-point grid sampling. The VESTA and X-Crysden programs were used to draw the molecular structure and visualize the electronic results.

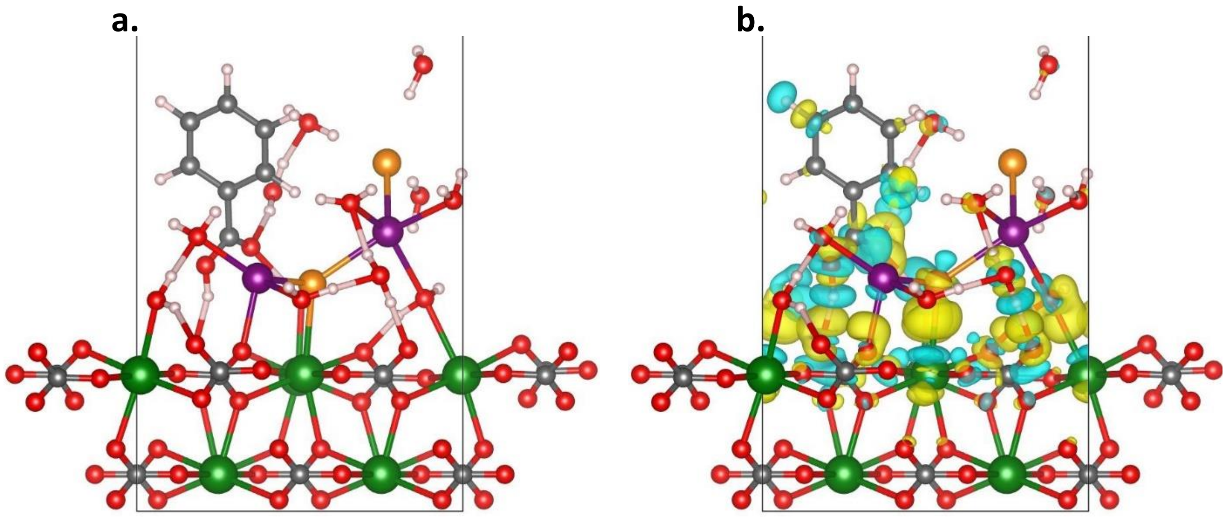

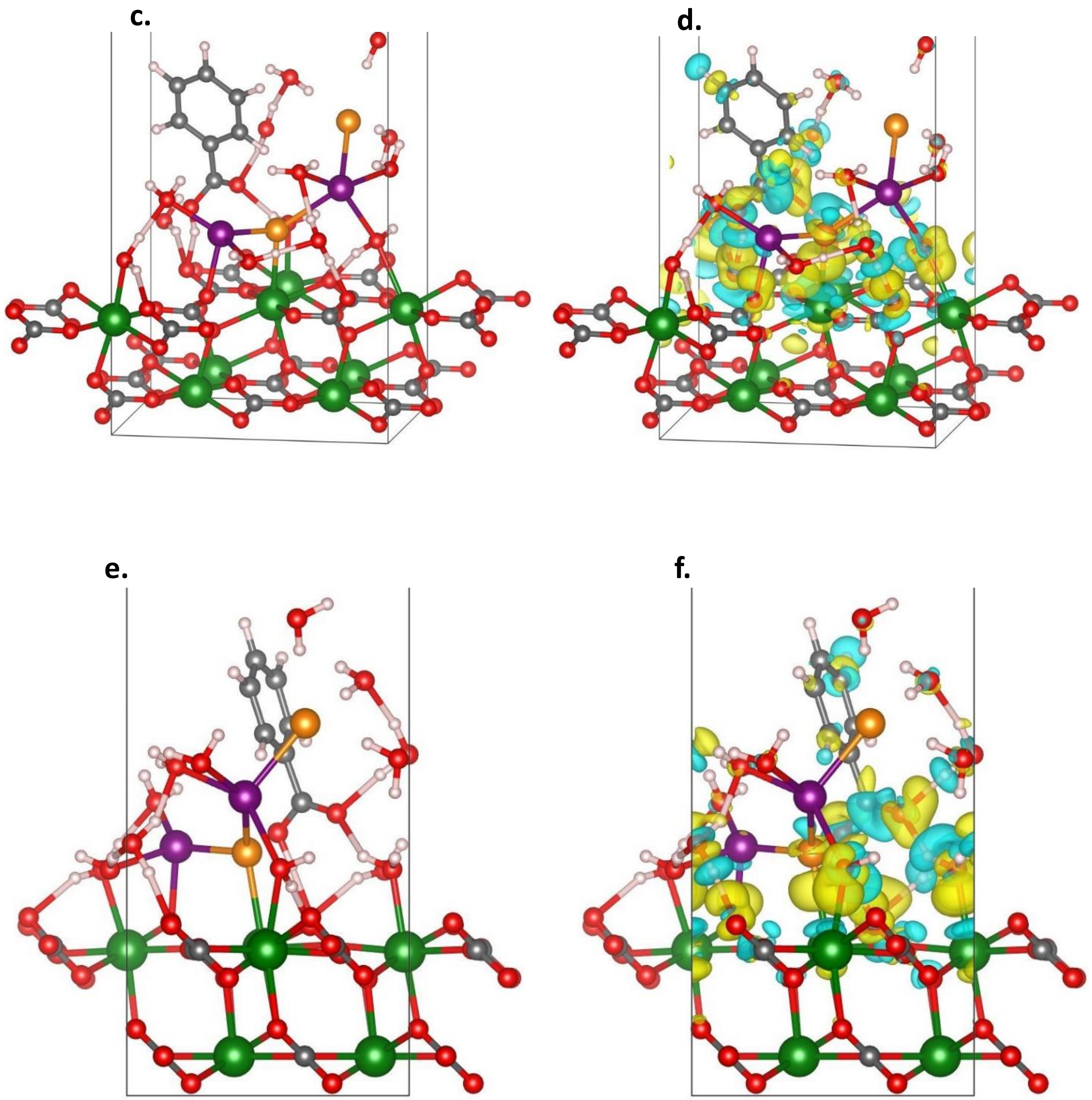

Figure S15. Left panels: Optimized configurations obtained by DFT calculation depicted in three different directions. Right panels: Charge density difference plot superimposed on optimized configurations. Electron accumulation and depletion are represented by yellow and blue areas, respectively. The atomic color codes are similar to those in other figures in the article except chlorides which are shown in orange to avoid confusion with charge density plots.
